# Safety evaluation of arabinase (arabinan endo‐1,5‐α‐L‐arabinanase) from *Aspergillus tubingensis*

**DOI:** 10.1002/fsn3.1329

**Published:** 2019-12-17

**Authors:** Nobuo Okado, Mai Sugi, Sawako Kasamoto, Fukutaro Mizuhashi, Ashley Roberts, Barbara Danielewska‐Nikiel, Christina Sulaiman, Sa Pham

**Affiliations:** ^1^ Shin Nihon Chemical Co., Ltd Aichi Japan; ^2^ Biosafety Research Center, Foods, Drugs, and Pesticides (currently BioSafety Research Center Inc.) Shizuoka Japan; ^3^ Intertek Scientific and Regulatory Consultancy Mississauga ON Canada

**Keywords:** Arabinase, *Aspergillus tubingensis*, food safety, genotoxicity, subchronic toxicity

## Abstract

Arabinase is an enzyme recognized for its ability to degrade arabinan, a plant cell wall constituent. It has been applied in the food industry most commonly for juice processing. One commercial source of arabinase is *Aspergillus tubingensis* (*A. tubingensis*), a black *Aspergillus* species. Given the intended use in food for human consumption, and noting its potential presence at trace levels in finished products, a series of safety studies including in vitro Ames and chromosome aberration assays, in vivo mammalian erythrocyte micronucleus and alkaline comet assays, and a 90‐day rat oral toxicity study were conducted. No test article‐related mutagenic activity was observed in the Ames assay. Although positive activity was observed in the chromosome aberration assay, this was not replicated in the in vivo genotoxicity assays including in preabsorptive cells. In the subchronic toxicity study, no test article‐related adverse effects were observed following oral administration of arabinase at doses of 15.3, 153, or 1,530 mg total organic solids (TOS)/kg body weight/day to Sprague Dawley rats. The no‐observed‐adverse‐effect level was considered to be the highest dose tested (1,530 mg TOS/kg body weight/day). The results of the genotoxicity studies and the subchronic toxicity study support the safe use of arabinase from *A. tubingensis* in food production.

## Graphical abstract

A series of toxicological studies including in vitro and in vivo short‐term genotoxicity assays and a 90‐day subchronic oral toxicity study were conducted to evaluate the safety of arabinase from *Aspergillus tubingensis*. Arabinase was concluded to be nongenotoxic and was not associated with toxicity in rats at the highest dose tested (up to 1,530 mg TOS/kg body weight/day). Study results provide reassurance of the safety of use of arabinase from *A. tubingensis* in food production.

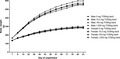

## 1 INTRODUCTION

Arabinase (5‐α‐L‐arabinan 5‐α‐L‐arabinanohydrolase; arabinan endo‐1,5‐α‐L‐arabinanase; EC 3.2.1.99), also referred to as arabanase or arabinanase, is a well‐characterized enzyme that catalyzes the endohydrolysis of (1 → 5)‐α‐arabinofuranosidic linkages in polysaccharides of arabinose (BRENDA Professional, 2018). Arabinose polysaccharides, also commonly known as arabinans, are constituents of plant cell walls. In 1967, Kaji and colleagues purified arabinase produced by *Aspergillus niger* (*A. niger*) and studied its properties, determining that the enzyme was responsible for the degradation of beet arabinan to L‐arabinose (Kaji, Tagawa, & Matsubara, 1967). The enzyme belongs to the broader group of pectinases, which are generally recognized for their use in the degradation of plant cell wall polysaccharides and as such have applications in fruit and vegetable processing, as well as juice and wine production (Aehle, 2007; Duvetter et al., 2009; OIV, 2018). In commercial practices, arabinase is typically used in fruit juice processing, particularly in the production of apple and pear juices, for its ability to break down arabinans, the formation of which is responsible for fruit juice cloudiness (Aehle, 2007; Bayindirli, 2010; Sharma, Patel, & Sharma, 2014; Voragen, Rombouts, Searle‐van Leeuwen, Schols, & Pilnik, 1987).

Commercially used arabinase can be derived from fungal sources, commonly the *Aspergillus* species (Sharma, Patel, & Sharma, 2014), although other microorganisms (e.g., *Bacillus subtilis*) also are known to produce the enzyme (Weinstein & Albersheim, 1979). *A. niger*‐derived “endo‐arabinase” is permitted for use in food in Australia/New Zealand as a processing aid (FSANZ, 2019). “Arabinanase” from *A. niger* also is included in the Codex Inventory of Substances Used as Processing Aids (Codex, 2018) and has been listed by the Association of Manufacturers and Formulators of Enzyme Products on the list of commercially used enzymes (Amfep, 2009), as well as by the International Organisation of Vine and Wine among enzymes used in wine production (OIV, 2012, OIV 2018). *Aspergillus tubingensis* (*A. tubingensis*)*,* a filamentous fungi species, which also belongs to *Aspergillus* section *Nigri* (the black aspergilli) (Samson, Houbraken, Kuijpers, Frank, & Frisvad, 2004), has been identified as a further producer of arabinase.

Morphologically, *A. tubingensis* is practically indistinguishable from *A. niger* (Susca, Stea, Mule, & Perrone, 2007), which is one of the most common microorganisms used for the production of food enzymes (Olempska‐Beer, Merker, Ditto, & DiNovi, 2006; Schuster, Dunn‐Coleman, Frisvad, & van Dijck, 2002). Distinction between *A. tubingensis* and *A. niger* can only be achieved by molecular methods (Susca et al., 2007), and until recently, the species could not be clearly differentiated (EFSA, 2009a). However, since the development of more advanced molecular methods, the “*A. niger* complex” has been divided into two separate species: *A. niger* and *A. tubingensis*. It may therefore be expected that in some cases where a microorganism was previously classified as *A. niger*, the production organism could in fact have been *A. tubingensis*, and thus, it is likely that at least some enzymes already used in food processing and thought to be products of *A. niger* strains are in fact produced by *A. tubingensis*.

Although arabinase already appears to have established commercial applications in the food industry, to our knowledge, no toxicological studies conducted to examine the safety of arabinase per se are available in the published literature. Evaluation of the safety of the production organisms also is a critical consideration in the safety evaluation of an enzyme intended for use in the production of food intended for human consumption (Pariza & Johnson, 2001). One study was identified in which the potential toxicity of a glucose oxidase from *A. tubingensis* (CTM 507) was examined (Kriaa, Mnafgui, Belhadj, El Feki, & Kammoun, 2015). Furthermore, although it may be possible that some arabinase enzyme preparations already in commercial use are products of *A. tubingensis* given as discussed the difficulties with the taxonomic classification of the *Aspergillus* section *Nigri*, this has yet to be confirmed, and thus at present, history of safe use for *A. tubingensis*‐derived arabinase is difficult to establish. Therefore, to confirm the safety of arabinase derived from *A. tubingensis*, and ascertain this *Aspergillus* species as a safe source for a food enzyme, key toxicity assessments were conducted in accordance with current study guidelines. The objective of the present study therefore was to specifically evaluate the safety of arabinase derived from a nongenetically modified *A. tubingensis* strain GPA41 (hereinafter referred to as AG) for use in food processing. To this end, AG, in the form of an ultrafiltered concentrate, was subjected to a standard battery of toxicological testing, consisting of a 90‐day repeated oral dose toxicity test conducted in rats and a series of in vitro and in vivo genotoxicity tests. The mutagenic potential of the AG concentrate was assessed in a bacterial reverse mutation assay, and an in vitro chromosome aberration test and an in vivo mammalian erythrocyte micronucleus test were used to evaluate the potential of the enzyme concentrate to induce chromosomal damage. The potential of AG concentrate to induce DNA damage was further examined in an in vivo comet assay conducted in rats.

## 2 MATERIALS AND METHODS

All studies were conducted at Biosafety Research Center (formally, Biosafety Research Center, Foods, Drugs, and Pesticides, currently BioSafety Research Center Inc.). Biosafety Research Center is a Good Laboratory Practice (GLP)‐compliant testing facility. All studies were performed in compliance with the Organisation for Economic Co‐operation and Development (OECD) Principles of Good Laboratory Practice (as revised in 1997) (ENV/MC/CHEM(98)17 – OECD, 1998a).

### 2.1 Test article

The AG ultrafiltered concentrate, produced as described below, was provided by Shin Nihon Chemical (Anjo) as a clear, brown‐colored, odorless liquid. The same manufacturing lot (Lot No. 120127T) was used in all studies. Enzymes derived from microbial sources usually are used in food in the form of enzyme preparations and as such typically include not only the enzyme responsible for the intended activity, but also other metabolites of the production strain, as well as formulation aids (Pariza & Johnson, 2001; U.S. FDA, 2010). The test article used in all studies met the established product specifications, including minimum required levels of enzyme activity, as well as current purity limits established for food enzyme preparations in the latest edition of the Food Chemicals Codex (FCC, 2018) and by the Joint FAO/WHO Expert Committee on Food Additives (JECFA, 2006), based on analytical testing. The ultrafiltered enzyme concentrate that was used did not contain any diluents, stabilizers, or preservatives. The AG enzyme concentrate was characterized by an arabinase activity of 690 unit (U)/mL and contained 153 mg total organic solids (TOS) per mL. The concept of expressing doses of enzyme applied in toxicological studies on the basis of TOS content, defined as 100% ‐ (A + W+D), where A = % ash, W = % water, and D = % diluents and/or other formulation ingredients, has been widely adopted in order to distinguish the proportion of the enzyme preparation derived from the source material and manufacturing process from that contributed by intentionally added formulation ingredients (JECFA, 1988; EFSA, 2009b). The enzyme concentrate was stored in an airtight container under frozen conditions (−27.6 to −16.2°C), and analytical testing conducted throughout the experimental periods of all studies, but the comet assay, indicated that the test article was enzymatically stable for the duration of the studies. In the case of the comet assay, the test article was assumed to be stable based on the expiry date provided for the test article by Shin Nihon and proper storage methods during the test period.

Briefly, the AG concentrate was prepared by standard culture methods using the production strain *A. tubingensis* GPA41. *A. tubingensis* strain GPA41 was originally isolated from a fruit source based on its ability to produce high arabinase activity, its viability, and its suitability for industrial production. The strain was identified as belonging to the species *Aspergillus tubingensis* by the Centraalbureau Voor Schimmelcultures (CBS‐KNAW Fungal Biodiversity Centre) using molecular methods (genomic analysis) consisting of gene amplification and sequencing and comparison to gene sequences in established DNA databanks, including NCBI nucleotide database (GenBank). The strain has been deposited at the National Institute of Technology and Evaluation (NITE)'s Biological Resource Center (NBRC) under No. NITE SD 00284.

Following cultivation, the *A. tubingensis* culture was subjected to multiple filtration and ultrafiltration steps to remove the production strain and other materials (e.g., proteins, saccharides, lipids, salts, and other compounds <5,000 molecular weight). A further series of filtration steps was then applied to remove insoluble materials and any potential contaminating microorganisms and residual amounts of the production strain to produce the liquid concentrate. For commercial purposes, the liquid concentrate may be formulated with glycerol to produce a liquid enzyme preparation. The concentrate was analytically determined to be free from any chemical (e.g., heavy metals and mycotoxins) or microbiological contamination (e.g., bacteria, including coliforms, *Escherichia coli*, and *Salmonella* species, and mold).

The enzyme concentrate as produced (153 mg TOS/mL) (Lot No. 120127T) served as the stock solution in all the studies. Test solutions of lower concentrations of arabinase activity were freshly prepared from the stock solution prior to use by serial dilution. With the exception of the chromosomal aberration assay, in which physiological saline (Japanese Pharmacopoeia, Otsuka Pharmaceutical Factory) was used as the solvent, in the case of all other testing, the stock solution was diluted with distilled water (Japanese Pharmacopoeia, Otsuka Pharmaceutical Factory).

### 2.2 Animals and general housing conditions used in the in vivo studies

Crl:CD(*SD*) rats were used in the 90‐day study and in the in vivo micronucleus and comet assays. Rats were purchased from the Charles River Laboratories Japan, Inc. and housed individually in wire mesh cages (19.7 × 26.3 × 18.0 cm) placed in an automatic water flushing breeding rack (Toyoriko) under the following conditions: 20.0 to 26.0°C (actual values: 22.7 to 23.2°C), relative humidity of 35 to 70% (actual values: 40.8 to 60.4%), 12 or more air changes per hour, and a 12‐hr light/dark cycle. Water [tap water (Iwata, Japan, tap water) from an automated water supply system] and food (commercial diet, CRF‐1; Oriental Yeast) were provided ad libitum. The feed and water were analyzed for contaminants, and levels in both were confirmed to be within the acceptable limits. The feeders were exchanged on a weekly basis.

### 2.3 90‐day oral toxicity study

The 90‐day repeated oral dose toxicity study was conducted in accordance with the OECD Guideline for the Testing of Chemicals No. 408 (OECD, 1998b) and the Guidelines for Designation of Food Additives and for Revision of Standards for Use of Food Additives (Ministry of Health, Labor and Welfare of Japan, 1996).

#### 2.3.1 Preparation of dosing formulations

The undiluted stock solution (153 mg TOS/mL) served as the high‐dose dose formulation (1,530 mg TOS/kg body weight). Lower doses of 15.3 (low dose) and 153 (mid‐dose) mg TOS/kg body weight/day were prepared by 10‐fold serial dilution of the thawed AG concentrate stock solution with distilled water to obtain concentrations of 1.53 and 15.3 mg TOS/mL, respectively. Stability of the dose formulations for low‐ and high‐dose groups was evaluated by determination of the enzyme activity, which was measured just after preparation of the dose formulations, after the formulations were stocked on ice for 4 hr, and after the formulations were stocked for 8 days under refrigeration and 24 hr at room temperature. The dose formulations were confirmed to be stable after being stored on ice for 4 hr, as well as after 8‐day storage in the refrigerator followed by 24‐hr storage at room temperature as no significant deviations were observed in enzyme activity compared to values obtained just after preparation of the dose formulations (see Table S1). New dose formulations were therefore prepared at intervals of not more than 8 days. Enzyme activity of the dose formulations was measured periodically throughout the study; acceptance criteria for enzyme activity of the dose formulations were met on every testing occasion.

#### 2.3.2 Animals and treatment

Forty‐five male and 45 female rats were purchased for the study. Upon receipt, the rats were 4 weeks old (±3 days). The animals were observed daily for general condition throughout the acclimation period. Animal body weights were measured on the day of receipt (Day −8) and at the end of the quarantine and acclimation period (Day −1). Throughout the 7‐day predosing period, no abnormalities were observed in the general condition or body weight gain. On the first day of dosing (Day 1), animals were randomly assigned to one of the four groups (10/sex/group), based on body weights using a computer system package for safety studies (LATOX‐F/V5, FFC). Prior to the initiation of the administration period (Day −2 and Day −1), ophthalmological examinations revealed unsuitable abnormalities among 1 male and 4 female rats; these animals were not assigned to a group (surplus animals). Body weights of the control animals and animals assigned to treatment groups ranged from 151 to 176 g for males and 122 to 147 g for females.

Doses of AG concentrate for the 90‐day study were selected based on a previous 2‐week repeated oral dose‐range finding rat toxicity study with the enzyme (unpublished data). AG concentrate doses of 15.3, 153, or 1,530 mg TOS/kg body weight/day (highest dose tested being the maximum achievable dose based on dosing volume of 10 mg/kg body weight and arabinase activity and TOS content of the test material of 690 U/mL and 153 mg TOS/mL, respectively) in the 2‐week study were not associated with any compound‐related adverse effects, and as such, 1,530 mg TOS/kg body weight/day (high‐dose) was selected as the maximum dose for the 90‐day study. The control group animals received the vehicle solvent, distilled water. The enzyme and the vehicle control were administered at a dosing volume of 10 mL/kg body weight once daily by oral gavage using a Teflon gastric tube and disposable syringe. Most recent individual animal body weights were used to calculate the actual dose formulation volumes. As described in Section 2.3.3, animal body weights were obtained on Day 1 (first day of dosing) and at 7‐day intervals thereafter. Mean group male and female body weights at each day that animals were weighed are presented in Table S2 (also see Section 3.1.1).

#### 2.3.3 Clinical observations, body weights, food consumption, and ophthalmology

All animals were observed for clinical signs of toxicity and mortality twice per day (before and after dosing), but for the day of necropsy (Day 91 or 92), when observations were recorded only once (before animals were euthanized). Animals were weighed prior to dosing every 7 days, starting on the first day of dosing (Day 1) until Day 90, as well as on the day of necropsy (terminal body weight). Body weight gain from Day 1 to Day 90 was calculated. Food provided to animals was weighed and was supplied weekly beginning on Day 1 (same day as determinations of body weights were made); amount of food remaining was weighed on the day of the next body weight measurement and mean daily food consumption (g/day) was calculated. Ophthalmological examinations were conducted on all the animals before initiation of dosing (Days −2 and −1) and on Day 89 on all surviving animals in the control and high‐dose groups. Eyes were examined for appearance and light reflex of the pupil, followed by pupil dilation (Mydrin P®, Santen Pharmaceutical, Lot No. M376261) for examination of the anterior part of the eyeballs, optic media, and fundus oculi.

#### 2.3.4 Clinical pathology and urinalysis

Blood was drawn for hematology and blood chemistry examinations from all surviving animals on the day of scheduled necropsy (Days 91 and 92). Animals were fasted prior to blood sampling, and blood samples were collected from the abdominal aorta under isoflurane anesthesia prior to necropsy. Blood samples required for hematological analysis were collected into tubes containing an anticoagulant [ethylenediaminetetraacetic acid dipotassium salt (EDTA‐2K)]. The blood samples were analyzed with a Hematology System (ADVIA120, Bayer), and the following parameters were analyzed: hematocrit, hemoglobin, red blood cell count, mean corpuscular volume, mean corpuscular hemoglobin, mean corpuscular hemoglobin concentration, reticulocyte ratio, reticulocyte count, platelet count, white blood cell count, differential leukocyte ratios, neutrophil count, lymphocyte count, monocyte count, eosinophil count, basophil count, and large unstained cell count.

To obtain plasma for blood coagulation analysis, additional blood samples were collected into tubes containing an anticoagulant (3.2% sodium citrate solution) and centrifuged at 1,700 *g* for 13 min at room temperature. Plasma samples were analyzed with a coagulation analyzer (STA Compact, Roche) for the following parameters: prothrombin time, activated partial thromboplastin time, and fibrinogen concentration.

For blood chemistry examinations, blood samples were collected into tubes containing a gel and clot activator (Venoject II, Terumo) and centrifuged at 1,700 *g* for 7 min at room temperature to obtain sera. The following parameters were analyzed using an automatic analyzer (Hitachi 7170, Hitachi): total protein, glucose, triglyceride, total cholesterol, blood urea nitrogen, creatinine, total bilirubin, aspartate aminotransferase, alanine aminotransferase, alkaline phosphatase (ALP), γ‐glutamyl transpeptidase, calcium, and inorganic phosphorus. The samples were also analyzed using an electrolyte analyzer (EA06R, A & T) to measure sodium, potassium, and chloride parameters. An electrophoresis analyzer (Epalyzer 2 plus, Helena Laboratories) was used to analyze for the following additional parameters: albumin ratio, α_1_‐globulin ratio, α_2_‐globulin ratio, β‐globulin ratio, γ‐globulin ratio, A/G ratio, albumin concentration, α_1_‐globulin concentration, α_2_‐globulin concentration, β‐globulin concentration, and γ‐globulin concentration.

Urine samples were collected overnight, on Days 85 and 86 of dosing from the same animals as those from which blood samples were drawn. Animals were fasted and deprived of water for the duration of the 17‐hr collection period. Prior to urine collection, 20 mL/kg body weight (based on most recent body weight) of tap water was provided to the animals by oral gavage. At the end of the urine collection period, the volume of total urine collected and color of the urine samples were determined. Ames test strips (N‐Multistix SG‐L, Siemens Healthcare Diagnostics) and an automatic strip reader (CLINITEK 500, Bayer) were used to analyze for the following parameters: pH, occult blood, ketone bodies, glucose, protein, bilirubin, and urobilinogen. Concentrations of electrolytes (sodium, potassium, and chloride) in the supernatant of urine samples centrifuged at about 400 *g* for 5 min were determined using an electrolyte analyzer (EA 06 R); osmotic pressure was determined using AUTO&STAT^TM^ OM‐6030. Total amounts of urinary electrolytes excreted also were calculated based on urinary volume. Urinary sediments were stained by the Sternheimer method and examined microscopically.

#### 2.3.5 Pathology

Following collection of blood samples, animals scheduled for necropsy were euthanized by exsanguination under isoflurane anesthesia. The pathological examination consisted of organ weight measurement, macroscopic examination (necropsy), and histopathological examination. Gross necropsy consisted of examination of the external surface of the body and orifices, as well as of the organs and tissues in the abdominal, thoracic, pelvic, and cranial cavities. The weights of the following organs of all surviving animals were measured, and the organ‐to‐body weight (based on body weights on the day of necropsy) ratios (“relative organ weights”) were calculated: heart, liver, spleen, kidneys, adrenal glands, prostate (including urethra), testes, ovaries, brain, pituitary gland, thymus, uterus, thyroid gland (including parathyroid glands), and seminal vesicle (including coagulation gland). Organs and tissues consistent with those specified in the OECD guidelines and the Guidelines for Designation of Food Additives and for Revision of Standards for Use of Food Additives were preserved for histopathological examination from all animals in all groups. The testes and eyes (including optic nerves and Harderian glands) were prefixed in formalin–acetic acid solution and Davidson's solution, respectively, prior to fixation in 10 vol% neutral buffered formalin solution. All other organs were fixed in an adequate volume of 10 vol% neutral buffered formalin solution. The lungs were fixed by dropping infusion of fixative. With the exception of the Zymbal's glands and nasal cavity that were preserved but not subjected to histopathological examination [Zymbal's gland of one high‐dose female with a nodule identified at gross pathology (see Section 3.1.3.) was examined], all other fixed tissue samples were embedded in paraffin, sectioned, and stained with hematoxylin and eosin in preparation for histopathology. Only tissue specimens from the control and high‐dose groups were subject of the histopathological examination; as no test article‐related findings were observed in the high‐dose group animals, histopathological examination was not conducted on tissue specimens from the low‐ and mid‐dose groups. Type and severity of the histopathological findings were recorded.

#### 2.3.6 Statistical analysis

Bartlett's test for homogeneity of variances was used to conduct statistical analysis for the following parameters: body weight, body weight gain, food consumption, hematology, blood chemistry, and urinalysis (volume, osmotic pressure, and electrolytes) values, and absolute and relative organ weights. Homogenous data (based on results of Bartlett's test) were analyzed by Dunnett's multiple comparison test to determine significant differences between the control group and each test substance‐treated group. Heterogenous data (based on results of Bartlett's test) were subjected to Steel's test to identify significant differences between the control group and each test article‐treated group. Fisher's exact test was used to statistically analyze the survival rates between the control group and each test article‐treated group, while data from the histopathological examination were analyzed using Wilcoxon's test using exact probability calculation. Significant differences were analyzed at the 5% level of significance in Bartlett's test and the 5% and 1% levels of significance in other tests using a two‐sided analysis.

### 2.4 Genotoxicity studies

#### 2.4.1 Bacterial reverse mutation test (Ames test)

The bacterial reverse mutation test was performed in accordance with OECD Guideline No.471 (OECD, 1997a) using the official preincubation method and a modified preincubation method involving a washing step to remove free amino acids released into the culture medium, also referred to as the “treat‐and‐wash” method.

Tester organisms used in the Ames assay included *Salmonella typhimurium* strains TA100, TA1535, TA98, and TA1537 (provided by Dr. Bruce N. Ames, University of California) and *Escherichia coli* WP2*uvrA* (provided by the National Institute of Hygienic Sciences, Japan currently the National Institute of Health Sciences, Japan). Distilled water served as a negative control for all tester organisms. The following compounds were used as positive controls in studies conducted with the preincubation method without metabolic activation: 2‐(2‐furyl)‐3‐(5‐nitro‐2‐furyl) acrylamide (AF‐2) (Wako Pure Chemical Industries, Lot No. STQ3987) at 0.01 µg/plate for TA100 and WP2*uvrA* and at 0.1 µg/plate for TA98, 0.5 µg/plate of sodium azide (Wako Pure Chemical Industries, Lot No. YCR0650) for TA1535, and 80 µg/plate of 9‐aminoacridine hydrochloride (9‐AA) (Sigma‐Aldrich, Lot No. 1126KD) for TA1537. In assays conducted with the same method in the presence of metabolic activation, 2‐aminoanthracene (2‐AA) (Wako Pure Chemical Industries, Lot No. KWL1226) was used as the positive control at the following concentrations: 0.5 µg/plate for TA98, 1.0 µg/plate for TA100, 2.0 µg/plate for TA1535 and TA1537, and 10 µg/plate for WP2*uvrA*. In studies using the modified (treat‐and‐wash) method, the following compounds were used as positive controls in assays without metabolic activation: 0.1 µg/plate of AF‐2 for TA98 and TA100, 2.0 µg/plate of 4‐nitroquinoline 1‐oxide (4‐NQO) (Tokyo Chemical Industry, Lot No. N4RDF) for TA1535, and 20 µg/plate of 9‐AA for TA1537. In assays conducted with the same method in the presence of metabolic activation, 2‐AA was used at the following concentrations: 1.0 µg/plate for TA98, 2.0 µg/plate for TA100, and 4.0 µg/plate for TA1535. Microsomal fractions (S9) isolated from the livers of phenobarbital‐ and 5,6‐benzoflavone‐induced male Sprague Dawley rats were used for metabolic activation purposes.

To establish appropriate concentrations for the study and to provide a preliminary mutagenicity evaluation, a “preliminary” and a concentration‐finding assay were conducted using the preincubation method. As a result of negative findings in WP2*uvrA* in both the absence and presence of S9, as well as in TA98 and TA1537 in the presence of S9 in the concentration‐finding study, these tester strains were tested in the main study under conditions of the preincubation method. The main study with the remaining organisms (TA100 and TA1535 with and without metabolic activation and TA 1537 without metabolic activation) was conducted using the modified (treat‐and‐wash) method. Since positive results were obtained in the main preincubation study in TA98 (with S9), TA98 was tested with and without S9 using the treat‐and‐wash method. The treat‐and‐wash method also was used to conduct an additional confirmatory study (to confirm the results of the main study conducted under conditions of the modified method).

The preliminary study was performed at final AG concentrate concentrations (prepared from the stock solution by serial dilution) of 0.000153, 0.00153, 0.0153, 0.153, 1.53 and 15.3 mg TOS/plate in the absence and presence of S9 (one plate per concentration). A positive control was not used in the preliminary study. The concentration‐finding study was performed at final AG concentrate concentrations of 0.063, 0.19, 0.57, 1.7, 5.1, and 15.3 mg TOS/plate in TA98, TA100, TA1535, and TA1537 and in WP2*uvrA* in the absence and presence of S9. Additional testing was conducted in TA1537 at lower AG concentrate concentrations of 0.00078, 0.0023, 0.0070 and 0.021 mg TOS/plate in the absence of S9.

The main studies, following either the preincubation or modified (treat‐and‐wash) methods, and the confirmatory study using the modified method were conducted with final AG concentrate concentrations of 0.96, 1.9, 3.8, 7.7, and 15.3 mg TOS/plate. WP2*uvrA* in the absence and presence of S9 and TA98 and TA1537 in the presence of S9 were tested in the main assay using the preincubation method. An additional, lower concentration of 0.48 mg TOS/plate was tested in TA98 in the presence of S9 in the main preincubation assay. Under the conditions of the modified preincubation (treat‐and‐wash) method, TA100, TA1535, and TA98 in the absence and presence of S9 and TA1537 in the absence of S9 were tested, and all tester strains in the main study using the treat‐and‐wash method were additionally exposed to a lower concentration of 0.48 mg TOS/plate. All assays in the concentration‐finding study, the main studies, and the confirmatory study were performed in triplicate.

All plates were observed macroscopically for precipitation of the test article at the start of treatment and the time of colony counting. Growth inhibitory effects were examined using a stereoscopic microscope, and revertant colonies were counted with an automated colony analyzer (CA‐11, System Sciences). A response was regarded as positive if the mean number of revertant colonies was two times greater than in the negative control, and a concentration‐dependent relationship was observed or the effects were reproducible. Statistical analysis of the data was not conducted.

#### 2.4.2 In vitro mammalian chromosome aberration test

The in vitro mammalian chromosomal aberration test was conducted in accordance with OECD Test Guideline No. 473 (OECD, 1997b) under the conditions of a 6‐hr short‐term assay and a 24‐hr continuous assay. Testing was conducted in cultured Chinese hamster lung (CHL) fibroblast cells supplied by the National Institute of Hygienic Sciences, Japan (currently the National Institute of Health Sciences, Japan).

S9 fractions isolated from the livers of male Sprague Dawley rats induced by phenobarbital and 5,6‐benzoflavone were used for metabolic activation purposes. Physiological saline served as the negative control. Mitomycin C (Kyowa Hakko Kirin, Lot No. 566ABB) was used as the positive control in the short‐term assay in the absence of S9 activation at a concentration of 0.1 µg/mL and in the continuous treatment assay at 0.05 µg/mL. Cyclophosphamide (Shionogi, Lot No. 4366) served as the positive control in the short‐term assay in the presence of S9 activation at a concentration of 12.5 µg/mL.

The short‐term assay was conducted in CHL cells incubated with AG concentrate for 6 hr at a final concentration of 6.3, 7.8, 9.8, 12.3, or 15.3 mg TOS/mL in the absence of S9 activation and 5.0, 6.3, 7.8, 9.8, 12.3, or 15.3 mg TOS/mL in the presence of S9 activation. The continuous assay was conducted in CHL cells incubated with AG concentrate for 24 hr at a final concentration of 5.0, 6.3, 7.8, 9.8, 12.3, or 15.3 mg TOS/mL in the absence of metabolic activation. The concentrations were selected based on results of preliminary testing to determine mitotic index and cell growth inhibition (data not shown).

An increase in the level of c‐mitosis, accompanied by a lack of mitotic cells, which was observed at all tested concentrations in the continuous treatment assay, precluded microscopic evaluation of metaphase cells for chromosomal aberrations (see below in “Results” section), and as such, an additional test was conducted using the continuous treatment protocol and lower AG concentrate concentrations (0.24, 0.40, 0.66, 1.1, 1.8, 3.1, or 5.1 mg TOS/mL).

Each culture was macroscopically examined for precipitation of the test article and any changes in color of the medium at the start and end of treatment.

All tests were conducted in duplicate. Cells were suspended in culture plates and incubated for 3 days. Cells were then treated with the solvent (negative control), AG concentrate, or positive control, and incubated for 6 hr for the short‐term assay and 24 hr for the continuous assays. In the short‐term assay, cells detached following the 6‐hr exposure to AG concentrate at concentrations of 12.3 mg TOS/mL or greater in the absence of S9 activation and at 15.3 mg TOS/mL in the presence of S9 activation. Cells were therefore isolated by pipetting, and all portions of the cell suspension were transferred to a centrifuge tube. Following centrifugation, at 1,000 r/min for 5 min, the supernatant was removed, and the cells were washed with 3 mL of Dulbecco's phosphate‐buffered saline (PBS). The supernatant was removed, and the cells were resuspended in medium, and returned to the original plates and incubated for a further 18 hr (total incubation of 24 hr after the start of exposure).

To inhibit the cells in the metaphase of the mitotic cycle, cells were exposed to colcemid solution (Life Technologies) at a final concentration of 0.2 µg/mL, 2 hr prior to preparation of the chromosome slides. In the continuous assays, cells also detached following exposure to AG concentrate at 12.3 mg TOS/mL or greater; cells were isolated by pipetting, and all portions of the cell suspension were transferred to a centrifuge tube and 2 mL of 0.25% trypsin solution (Life Technologies) was added to the tube. In all other cases, the mediums were transferred to the centrifuge tube, following which the cells were detached from the plates with 2 mL of 0.25% trypsin solution, and the cell suspensions were transferred to the tube.

A portion of the cell suspension was removed for determination of cell growth inhibition rates. An automated cell counter (TC20™, Bio‐Rad) was used to count the number of cells per volume, and the relative growth rate was calculated using the ratio of the number of cells of each concentration to the negative control. The remaining cell suspension was centrifuged (1,000 r/min for 5 min), the supernatant was removed, and the cells were treated with 5 mL of 75 mmol/L warmed potassium chloride solution, which was again followed by centrifugation and removal of supernatant. The cells were fixed in an ice‐cold solution of methanol and acetic acid (3:1). One slide per plate was prepared to confirm the cell density, and two slides per plate were prepared for chromosome analysis. The slides were stained with 1.2% Giemsa solution. The lowest concentration that resulted in less than 50% relative cell growth rate or the analyzable limit concentration (in the case of the additional continuous treatment assay) was selected as the highest concentration to be included for microscopic evaluation, in addition to the two consecutive lower concentrations. For each concentration selected for microscopic evaluation, 100 metaphase cells per plate were examined microscopically for the following chromosomal or chromatid‐type aberrations: chromosome and chromatid gaps, breaks, exchanges, and others. The number of polyploid cells (38 chromosomes or more) also was counted in 200 metaphases for each concentration.

The significance of the incidence of aberrant cells relative to the negative control group was analyzed using a one‐tailed Fisher's exact test with a 2.5% level of significance. A one‐sided Cochran–Armitage trend test with a 2.5% significance level was used to determine the presence of a concentration‐dependent relationship in statistically different groups. A response was considered as positive if the incidence of aberrant cells in the treated groups was significantly greater relative to the negative control group and a significant concentration‐dependent relationship was established, or if the results were reproducible. The final evaluation was performed on the total incidence of aberrant cells minus the number of cells with only gaps.

In order to compare the clastogenic potential of AG to known clastogens, the D_20_ value, defined as a concentration of the test article that causes anomalies in 20% of the metaphase cell, was calculated by the method of least squares. Additionally, the translocation (TR) value, an index for the chromatid exchange incidence per concentration (mg/mL) of the test article, also was determined.

#### 2.4.3 In vivo mammalian erythrocyte micronucleus test

The in vivo mammalian erythrocyte micronucleus test was conducted in accordance with OECD Guideline for the Testing of Chemicals No. 474 (OECD, 1997c). Thirty‐three male 7‐week‐old rats were purchased and, following receipt at the laboratory, monitored daily for general condition during a 1‐week quarantine and acclimation period. Body weight was measured at the beginning of the acclimation period (Day −7, receipt of animals) and on Day 1 (grouping of animals and first day of dosing). No abnormalities were observed in general condition and body weight gain. The animals were stratified by body weight and randomly assigned to one of the five groups (6 rats/group) on Day 1.

Since no noteworthy toxic findings or sex differences were observed in a previous 2‐week repeated oral dose toxicity study with AG concentrate administered to rats at 1,530 mg TOS/kg body weight (unpublished data), a maximum dose of 1,530 mg TOS/kg body weight was selected for the in vivo micronucleus assay, and only male rats were used for the experiment. Aqueous solutions of 38.3 and 76.7 mg TOS/mL were prepared by twofold serial dilution of the stock solution (153.0 mg TOS/mL) with distilled water to obtain final doses of 383 (low‐dose), 767 (mid‐dose), and 1,530 (high‐dose) mg TOS/kg body weight. The test material was administered at a volume of 10 mL/kg body weight once daily for two consecutive days (24‐hr interval) using a plastic syringe and Teflon gastric tube. The positive control group received a single dose of 10 mg/kg body weight of cyclophosphamide (CP) (Shionogi & Co., Lot No. 4366); CP was dissolved in physiological saline (Japanese Pharmacopoeia, Otsuka Pharmaceutical Factory, Lot No. K2E00) using a plastic syringe and Teflon gastric tube. The negative control group was administered the test article solvent (distilled water).

The general condition of test and negative control animals was observed at 1, 24, 25, and 48 hr after initial dosing. Additionally, animal body weights were measured immediately before animals were euthanized. The positive control animals were observed for general condition at 1 hr after dosing and prior to bone marrow preparation (24 hr after dosing). The animals were euthanized by inhalation of CO_2_ 24 hr after administration of the second dose and in the case of the positive control animals, 24 hr after dosing with CP. The femur was removed, and the bone marrow cells were flushed out with calf serum (Life Technologies, inactivated at 56°C for 30 min), centrifuged to remove excess serum (1,000 r/min, 5 min; centrifuge LC‐122, Tomy Seiko), and resuspended in Dulbecco's PBS (Sigma‐Aldrich). The resulting cell suspension was centrifuged once more and the supernatant removed, following which the entire procedure was repeated twice. The cells were fixed in 5 mL of 10% neutral buffered formalin solution (Wako Pure Chemical Industries). Following this, the fixative was exchanged twice by centrifugation, the cells were resuspended in formalin solution, and the cell suspension was filtered with a cell strainer (pore size 35 µm, Becton Dickinson Labware).

Fixed cell suspensions were prepared for all 6 animals per group and were coded at random. Microscopic examination of the cells was performed on 5 animals per group (in ascending order of animal identification number). The cell suspensions were placed on a coverslip and immediately put on a slide coated with acridine orange and analyzed microscopically using a fluorescent microscope (×800) equipped with a blue excitation filter and a barrier filter. Two thousand (2,000) immature erythrocytes (IE) per animal were analyzed, and the number of micronucleated immature erythrocytes (MNIE) was counted. The number of IE out of a total of 500 erythrocytes also was counted in order to examine the effect of the test article on bone marrow cell proliferation. The ratio of IE to analyzed (total) erythrocytes and the frequency of MNIE were calculated. The data on the frequency of MNIE were analyzed by conditional binomial test (Kastenbaum and Bowman test) using an upper‐tailed significance level of 2.5% to determine significant differences between the negative control group and each of the other groups (including the positive control group). The data on the ratio of IE to analyzed erythrocytes were subjected to Dunnett's multiple comparison test, using a two‐tailed significance level of 5%. An Aspin–Welch *t* test also was used to analyze the significance of differences between the negative and positive control group data, using a two‐tailed significance level of 5%. A result was determined to be positive when the difference in the frequency of MNIE between a treatment group and the negative control group was statistically significant.

#### 2.4.4 In vivo comet test

The in vivo mammalian alkaline comet assay was conducted in accordance with OECD Guideline for the Testing of Chemicals No. 489 (OECD, 2016). Thirty‐three rats, 7 weeks of age, were purchased and monitored daily for general condition during a 1‐week quarantine and acclimation period. Body weight was measured at the beginning of the acclimation period (Day −8, receipt of animals) and on Day 1 (grouping of animals and first day of dosing). No abnormalities in general condition and body weight gain were observed. At the end of the acclimatization period (Day 1), animals were stratified by body weight and assigned to one of the five groups (6 rats/group).

On the basis of a 2‐week repeated oral dose study of AG concentrate conducted in rats at a maximum dose of 1,530 mg TOS/kg body weight that resulted in no adverse effects or sex differences (unpublished data), only male rats were used and AG concentrate doses of 383, 765, and 1,530 mg TOS/kg body weight were selected for the comet assay. Aqueous solutions of 38.3 and 76.5 mg TOS/mL were prepared by twofold serial dilution of the stock solution (153 mg TOS/mL) with distilled water to obtain final doses of 383 (low dose), 765 (mid dose), and 1,530 (high dose) mg TOS/kg body weight. The test material was administered to rats *via* gavage using a plastic syringe and Teflon gastric tube. Ethyl methanesulfonate (Lot No. BCBN1209V; Sigma‐Aldrich) served as the positive control in the study at a dose of 200 mg/kg body weight. Distilled water, the test article solvent, was used as the negative control. Both the positive and negative control substances also were administered by gavage using plastic syringes and Teflon gastric tubes. The dosing volumes were 10 mL/kg body weight, and the animals were administered the solvent, the test article formulation, and the positive control substance once daily, for two consecutive days, at 21‐hr intervals.

Clinical observations were performed at 1, 21 (prior to the second administration), 22, and 24 (just prior to necropsy) hours following the first administration. Body weight measurements were obtained just before necropsy. The animals were euthanized by CO_2_ inhalation 3 hr after the second administration for resection of the glandular stomach and duodenum. The glandular stomach and duodenum were selected because of their initial exposure to the test article following oral administration and relatively high concentration of the test article in these organs.

Following removal, the glandular stomach and duodenum were rinsed with homogenizing buffer [Hanks’ balanced salt solutions (Life Technologies) containing 25 mmol/L EDTA·2Na (Dojindo Laboratories) and 10 v/v% dimethyl sulfoxide (DMSO) (Wako Pure Chemical Industries), pH 7.5] and macroscopically examined for any abnormalities. Next, the glandular stomach and duodenum were dissected, and rinsed with homogenizing buffer, and portions of each organ were collected for potential histopathology. In preparation for the comet assay, the remaining portion of the glandular stomach was incubated for 15 to 30 min in cooled homogenizing buffer. The surface epithelium was subsequently scraped gently several times using a blade and discarded. To release the cells, the stomach was placed in a dish containing homogenizing buffer and the epithelium was scraped gently several times using a blade. In the case of the duodenum, tissue samples were homogenized using a Dounce tissue grinder, after adding an adequate amount of homogenizing buffer to each tissue sample. The glandular stomach and duodenal cell suspensions were then placed in a tube and centrifuged (800 r/min, 5 min). Following the removal of an appropriate amount of supernatant, the cells were resuspended in the remaining supernatant and 10 µl of the cell suspensions was transferred into microtubes.

Slides were prepared in triplicates (two slides for evaluation, one as a spare) by adding 90 µl of 0.5% low‐melting agarose gel (Lonza Rockland, Lot No. 0000490188) to the microtubes containing the cell suspensions and mixed; 90 µl of the cell‐agarose mixture was placed onto a superfrosted glass slide, which had been precoated with 1.0% agarose (Type I, Low EEO, Sigma‐Aldrich, Lot No. SLBN6401V) and covered with a noncoated superfrosted glass slide. Once the agarose solidified, the covering glass slide was removed and another layer of 90 µl of 0.5% low‐melting agarose added in the same manner. The slides were placed in lysing solution [2.5 mol/L NaCl, 100 mmol/L EDTA‐2Na, 10 mmol/L, pH 10 Tris buffer, 10 v/v% DMSO and 1 v/v% Triton X‐100 (Sigma‐Aldrich)] and refrigerated overnight under light‐protected conditions.

The slides were rinsed using electrophoresis buffer [300 mmol/L NaCl (Kanto Chemical), 1 mmol/L EDTA·2Na (Dojindo Laboratories), pH > 13] and placed in a submarine‐type electrophoresis chamber (BE‐540, BIO CRAFT). Afterward, slides were immersed by the gentle addition of chilled electrophoresis buffer to the chambers and then left for 20 min (unwinding). Following unwinding, electrophoresis was performed for 20 min at a constant voltage of 0.7 V/cm (25V) (initial current: 300 mA). In order to maintain the stability of the electrophoresis buffer, the electrophoresis chambers were cooled on ice during unwinding (at the start: 0.1 to 0.5°C) and electrophoresis (at the start: 1.1 to 2.0°C; at the end: 3.8 to 4.9°C). The slides were subsequently immersed in neutralizing solution [0.4 mol/L tri (hydroxymethyl) aminomethane; pH 7.5] for 10 min to neutralize the alkali in the gels. Following dehydration of the slide in ethanol (≥99.5%) for 10 min, slides were examined for DNA damage. As no damage to any of the slides occurred during slide preparation, the spare slides were not electrophoresed.

Dehydrated slides were randomly coded and then treated with 50 µl of SBYR® Gold nucleic acid gel stain (Life Technologies) diluted 5,000‐fold with Tris‐EDTA buffer (pH 8.0, Nippon Gene). Slides from 5 animals per group (in ascending order of animal identification number) were examined using a fluorescent microscope (Olympus Life Science Solutions). One hundred and fifty cells per animal (75 cells per slide; 750 cells per group of 5 animals) per organ were analyzed. Images of DNA migration in cells were imported to a computer *via* a charge‐coupled device camera connected to the microscope and analyzed using comet assay analyzer software (Comet Assay IV System, Perceptive Instruments). Additionally, the number of hedgehogs was counted in a further 150 cells per animal (75 cells per slide) using the fluorescent microscope. Histopathological examination of the organs was not conducted due to the absence of DNA damage in either of the sampled organs in the comet assay.

DNA damaged was determined as the percentage of tail DNA compared to the total (% tail DNA: Tail % intensity). The mean % tail DNA (animal value) was calculated from the median % tail DNA for each slide (slide value). Significance of differences in mean % tail DNA of animals in the test groups relative to the negative control group was analyzed using Dunnett's multiple comparison test (two‐sided) with a 5% level of significance. For comparison of the mean % tail DNA of the positive control group relative to that of the negative control group, statistical analysis was conducted by Aspin–Welch's *t* test (one‐sided) with a 2.5% level of significance. A result was determined to be positive when the difference in the % tail DNA between a treatment group and the negative control group was statistically significant, a significant dose‐dependency was established, and the % tail DNA in the test article group was outside the historical negative control data.

## 3 RESULTS

### 3.1 90‐day oral toxicity study

#### 3.1.1 Clinical observations, body weights, food consumption, and ophthalmology

**Figure 1:**
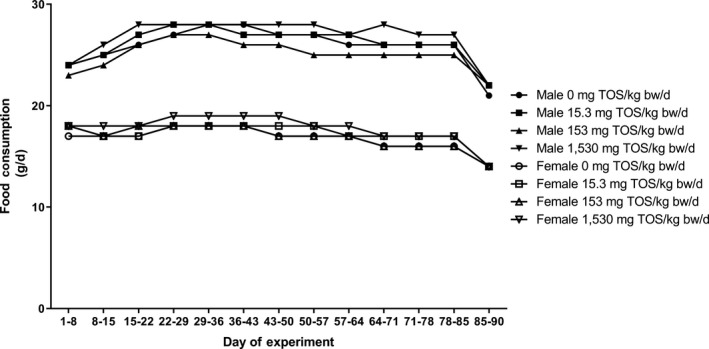
Food consumption of male and female rats in the 90‐day repeated oral (gavage) dose toxicity study on AG concentrate

**Figure 2:**
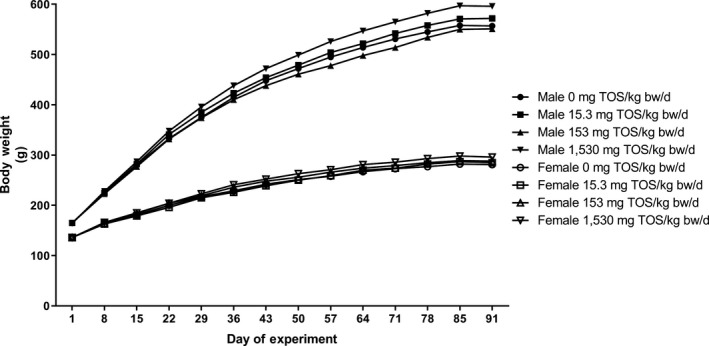
Body weights of male and female rats in the 90‐day repeated oral (gavage) dose toxicity study on AG concentrate

All control animals and those administered AG concentrate survived the course of the live phase of the study in good condition until scheduled necropsy. One male in the low‐dose group (15.3 mg TOS/kg body weight/day) experienced back trauma during Days 30 to 37, but recovered thereafter without any apparent residual symptoms. Food consumption (Figure 1) of test animals was comparable to that of the controls throughout the study period. Likewise, no statistically significant differences in body weight (Figure 2 and Table S2) or body weight gain were observed among the different groups of animals; however, end‐of‐treatment body weights and body weight gains attained over the course of the dosing period of high‐dose animals were higher than those of controls. Ophthalmological examinations revealed no test article‐related findings.

#### 3.1.2 Clinical pathology and urinalysis

Differences in hematological parameters between control and test group animals to reach statistical significance consisted of reductions in the large unstained cell ratio (*p* ≤ .05) and count (*p* ≤ .01), and in the fibrinogen level (*p* ≤ .05) in mid‐dose (153 mg TOS/kg body weight/day) males (see Table 1). As shown in Table 2, clinical chemistry analysis revealed statistically significantly higher potassium (4.59 ± 0.11 mmol/L vs. 4.35 ± 0.18 mmol/L; *p* ≤ .05), creatinine (0.37 ± 0.02 mg/dl vs. 0.33 ± 0.03; *p* ≤ .05), and ALP (353 ± 40 U/L vs. 291 ± 51 U/L; *p* ≤ .01) levels in high‐ (1,530 mg TOS/kg body weight/day), low‐ (15.3 mg TOS/kg body weight/day), and mid‐dose males, respectively. None of the hematological or clinical chemistry variations occurred in the presence of a clear dose–response and were only observed in males. Compared to the control group, no significant differences were noted in any of the examined urinalysis parameters of AG concentrate‐treated animals (data not shown).

**Table 1:** Hematology values for male and female rats administered AG concentrate by gavage for 13 weeks

Parameter measured (mean ± *SD*)	Dose group (mg TOS/kg body weight)							
	Males (*n* = 10)				Females (*n* = 10)			
	0 (control)^a^	15.3	153	1,530	0 (control)^a^	15.3	153	1,530
Hematocrit (%)	44.6 ± 1.5	44.4 ± 1.5	44.8 ± 0.9	43.3 ± 1.4	43.0 ± 0.8	43.0 ± 1.4	43.6 ± 1.1	42.6 ± 1.1
Hemoglobin (g/dL)	15.7 ± 0.6	15.7 ± 0.6	15.8 ± 0.3	15.3 ± 0.6	15.5 ± 0.3	15.5 ± 0.4	15.7 ± 0.6	15.4 ± 0.3
RBC (×10^6^/mm^3^)	8.49 ± 0.35	8.58 ± 0.33	8.59 ± 0.21	8.49 ± 0.43	8.00 ± 0.24	7.89 ± 0.24	8.08 ± 0.25	8.13 ± 0.18
MCV (µm^3^)	52.6 ± 1.8	51.8 ± 1.1	52.1 ± 1.5	51.0 ± 1.4	53.8 ± 1.4	54.5 ± 1.2	54.0 ± 1.9	52.4 ± 1.4
MCH (pg)	18.5 ± 0.8	18.3 ± 0.4	18.4 ± 0.6	18.0 ± 0.6	19.4 ± 0.7	19.7 ± 0.4	19.4 ± 1.0	19.0 ± 0.6
MCHC (%)	35.2 ± 0.5	35.3 ± 0.6	35.3 ± 0.5	35.3 ± 0.7	36.1 ± 0.7	36.1 ± 0.7	36.0 ± 0.7	36.2 ± 0.7
Reticulocyte (%)	2.2 ± 0.4	2.2 ± 0.2	2.1 ± 0.3	2.2 ± 0.2	1.8 ± 0.3	1.9 ± 0.3	1.9 ± 0.2	1.8 ± 0.2
Reticulocyte (×10^9^/L)	185.8 ± 32.8	190.6 ± 16.9	179.0 ± 25.0	191.1 ± 14.6	144.9 ± 26.4	151.9 ± 21.7	151.5 ± 17.9	145.1 ± 20.1
WBC (×10^3^/mm^3^)	10.60 ± 2.55	9.84 ± 3.23	8.38 ± 2.15	8.61 ± 1.53	5.11 ± 1.41	5.44 ± 1.64	4.85 ± 1.14	5.63 ± 1.65
Differential leukocyte ratios (%)								
Neutrophil	14.6 ± 4.5	14.6 ± 4.5	17.5 ± 8.2	14.1 ± 5.7	15.5 ± 3.3	14.7 ± 5.0	16.1 ± 5.1	19.7 ± 7.1
Lymphocyte	80.6 ± 5.0	80.8 ± 5.1	77.4 ± 8.6	80.4 ± 6.7	78.1 ± 3.2	79.6 ± 5.9	78.4 ± 5.3	74.4 ± 7.5
Monocyte	2.3 ± 0.5	2.3 ± 0.7	2.5 ± 0.8	2.7 ± 0.7	3.4 ± 0.9	2.8 ± 1.0	2.8 ± 0.7	3.1 ± 1.0
Eosinophil	1.6 ± 0.6	1.3 ± 0.4	2.0 ± 0.6	1.8 ± 0.6	2.2 ± 0.5	1.8 ± 0.6	1.7 ± 0.6	1.9 ± 0.7
Basophil	0.1 ± 0.1	0.1 ± 0.0	0.1 ± 0.1	0.1 ± 0.0	0.0 ± 0.1	0.1 ± 0.1	0.1 ± 0.0	0.1 ± 0.1
LUC	0.9 ± 0.3	0.9 ± 0.2	0.6 ± 0.2^b^	0.9 ± 0.3	0.8 ± 0.4N	1.1 ± 0.2	0.9 ± 0.6	0.8 ± 0.3
Differential leukocyte counts (×10^3^/mm^3^)								
Neutrophil	1.50 ± 0.47	1.37 ± 0.42	1.35 ± 0.38	1.22 ± 0.53	0.79 ± 0.27	0.78 ± 0.30	0.76 ± 0.22	1.05 ± 0.32
Lymphocyte	8.59 ± 2.29	8.02 ± 2.94	6.61 ± 2.24	6.93 ± 1.38	4.00 ± 1.16	4.36 ± 1.47	3.83 ± 1.06	4.24 ± 1.52
Monocyte	0.24 ± 0.08	0.22 ± 0.10	0.20 ± 0.06	0.23 ± 0.06	0.17 ± 0.05	0.14 ± 0.04	0.13 ± 0.04	0.18 ± 0.07
Eosinophil	0.16 ± 0.03	0.12 ± 0.04	0.16 ± 0.04	0.15 ± 0.04	0.11 ± 0.06	0.09 ± 0.03	0.09 ± 0.04	0.11 ± 0.04
Basophil	0.01 ± 0.01	0.01 ± 0.01	0.01 ± 0.01	0.01 ± 0.01	0.00 ± 0.00	0.00 ± 0.01	0.00 ± 0.01	0.00 ± 0.01
LUC	0.10 ± 0.04	0.09 ± 0.04	0.05 ± 0.02^c^	0.08 ± 0.02	0.04 ± 0.02	0.06 ± 0.02	0.04 ± 0.03	0.05 ± 0.02
Blood coagulation analysis								
Platelet (×10^3^/mm^3^)	1,028 ± 98	1,001 ± 121	987 ± 94	1,028 ± 76	1,013 ± 70	1,086 ± 132	1,054 ± 99	1,039 ± 83
PT (seconds)	12.6 ± 1.8	12.9 ± 2.1	13.0 ± 1.5	12.7 ± 1.5	8.8 ± 0.2N	8.8 ± 0.3	8.8 ± 0.2	8.9 ± 0.1
APTT (seconds)	25.7 ± 1.8	25.1 ± 2.4	26.0 ± 2.0	25.0 ± 2.2	17.4 ± 1.3	17.6 ± 1.7	17.4 ± 1.0	17.9 ± 1.3
Fibrinogen (mg/dl)	323 ± 25	301 ± 19	295 ± 22^b^	313 ± 27	226 ± 28	219 ± 14	229 ± 23	225 ± 29

Abbreviations: *A. tubingensis, Aspergillus tubingensis*; AG, arabinase from nongenetically modified *A. tubingensis* strain GPA41; APTT, activated partial thromboplastin time; LUC, large unstained cells; MCH, mean corpuscular hemoglobin; MCHC, mean corpuscular hemoglobin concentration; MCV, mean corpuscular volume; *N*, nonparametric analysis; PT, prothrombin time; RBC, red blood cell count; *SD*, standard deviation; TOS, total organic solids; WBC = white blood cell count.

a Control animals were administered distilled water.

b Significantly different from control (*p* ≤ .05).

c Significantly different from control (*p* ≤ .01).

**Table 2:** Selected blood chemistry values for male and female rats administered AG concentrate by gavage for 13 weeks

Parameter measured (mean ± *SD*)	Dose group (mg TOS/kg body weight)							
	Males (*n* = 10)				Females (*n* = 10)			
	0 (control)^a^	15.3	153	1,530	0 (control)^a^	15.3	153	1,530
BUN (mg/dl)	13.7 ± 1.2	14.5 ± 2.0	14.3 ± 2.1	15.3 ± 1.2	17.6 ± 2.4	18.0 ± 3.0	16.5 ± 1.7	17.1 ± 2.8
Creatinine (mg/dl)	0.33 ± 0.03	0.37 ± 0.02^b^	0.35 ± 0.03	0.34 ± 0.04	0.38 ± 0.06N	0.44 ± 0.13	0.38 ± 0.05	0.38 ± 0.06
AST (U/L)	68 ± 11	66 ± 11	68 ± 9	63 ± 9	68 ± 10N	81 ± 23	65 ± 12	74 ± 13
ALT (U/L)	26 ± 4	26 ± 2	27 ± 5	26 ± 3	26 ± 7	24 ± 5	22 ± 6	25 ± 9
ALP (U/L)	291 ± 51	306 ± 50	353 ± 40^c^	292 ± 24	174 ± 67N	162 ± 50	151 ± 30	172 ± 23
Calcium (mg/dl)	9.58 ± 0.22	9.41 ± 0.35	9.35 ± 0.15	9.56 ± 0.34	9.84 ± 0.28	9.74 ± 0.42	9.74 ± 0.23	9.55 ± 0.24
Inorganic phosphorus (mg/dl)	5.87 ± 0.38	5.64 ± 0.41	5.45 ± 0.45	5.83 ± 0.49	5.10 ± 0.70	5.13 ± 1.00	5.12 ± 0.87	4.80 ± 0.92
Sodium (mmol/L)	143.4 ± 0.7	143.2 ± 0.9	143.2 ± 0.5	142.9 ± 0.7	142.8 ± 1.5	142.6 ± 1.0	143.9 ± 0.9	142.8 ± 1.3
Potassium (mmol/L)	4.35 ± 0.18N	4.52 ± 0.29	4.48 ± 0.25	4.59 ± 0.11^b^	4.34 ± 0.33	4.57 ± 0.26	4.37 ± 0.27	4.24 ± 0.27
Chloride (mmol/L)	107.6 ± 0.7	107.7 ± 1.1	108.0 ± 1.3	106.5 ± 1.3	108.8 ± 2.1	109.4 ± 1.3	109.1 ± 1.5	109.3 ± 1.6

Note

*A. tubingensis*, *Aspergillus tubingensis*; AG, arabinase from nongenetically modified *A. tubingensis* strain GPA41.

Abbreviations: ALP, alkaline phosphatase; ALT, alanine aminotransferase; AST, aspartate aminotransferase; BUN, blood urea nitrogen; *N*, nonparametric analysis; *SD*, standard deviation; TOS, total organic solids.

a Control animals were administered distilled water.

b Significantly different from control (*p* ≤ .05).

c Significantly different from control (*p* ≤ .01).

#### 3.1.3 Pathology

Relative (to body weight) weights of the spleen (0.145 ± 0.010% vs. 0.168 ± 0.020%; *p* ≤ .05) and thymus (0.055 ± 0.015% vs. 0.073 ± 0.015%; *p* ≤ .05) were significantly lower in high‐dose males compared to controls, as were absolute spleen weights (0.76 ± 0.06 g vs. 0.89 ± 0.10 g; *p* ≤ .05) of mid‐dose males (Table 3). In females, absolute kidney weights were higher in the high‐dose group (1.92 ± 0.23 g vs. 1.69 ± 0.15 g; *p* ≤ .05) (Table 3). A clear dose–response was not apparent for any of the organ weight differences between control and test group animals. Macroscopic findings that did not have any co‐occurrence in the control group consisted of single incidences of a cyst in the spleen, a brown patch in the lungs, a brown and a red patch in the liver, and a dilated renal pelvis in males of the high‐dose group. In high‐dose females, a nodule in the stomach, a diverticulum in the ileum, and a nodule in the Zymbal's gland were detected. Several additional gross variations were detected in animals of the low‐ and mid‐dose groups without similar occurrences in the control group; however, the findings were detected singly and were not observed at the higher dose level(s). Histopathological observations that occurred in both males and females of the high‐dose group without any corresponding findings in the control group animals included edema in the glandular stomach (2 males and 2 females) and regeneration of acinar cells in the pancreas (2 males and 1 female). One case of adenoma of the Zymbal's gland also was identified in a high‐dose female (see Table S3).

**Table 3:** Absolute and relative organ weights of male and female rats administered AG concentrate by gavage for 13 weeks

Parameter measured	Dose group (mg TOS/kg body weight)							
	Males (*n* = 10)				Females (*n* = 10)			
	0 (control)^a^	15.3	153	1,530	0 (control)^a^	15.3	153	1,530
Absolute organ weight								
Brain (g)	2.29 ± 0.12	2.28 ± 0.10	2.30 ± 0.07	2.30 ± 0.09	2.06 ± 0.06	2.04 ± 0.08	2.03 ± 0.09	2.07 ± 0.09
Heart (g)	1.54 ± 0.15	1.60 ± 0.12	1.61 ± 0.23	1.62 ± 0.15	0.86 ± 0.09	0.89 ± 0.09	0.89 ± 0.11	0.95 ± 0.12
Liver (g)	14.03 ± 1.65	14.07 ± 1.44	13.28 ± 1.40	15.82 ± 2.18	6.30 ± 0.73	6.59 ± 0.84	6.47 ± 0.78	6.82 ± 0.95
Kidneys (g)	3.26 ± 0.29	3.29 ± 0.28	3.11 ± 0.26	3.42 ± 0.36	1.69 ± 0.15	1.82 ± 0.14^b^	1.77 ± 0.21	1.92 ± 0.23^d^
Spleen (g)	0.89 ± 0.10	0.86 ± 0.13	0.76 ± 0.06^d^	0.83 ± 0.10	0.47 ± 0.11	0.47 ± 0.06	0.47 ± 0.08	0.50 ± 0.09
Adrenal glands (mg)	62 ± 11	65 ± 12	56 ± 7	61 ± 11	61 ± 11	64 ± 9	60 ± 10	63 ± 13
Testes (g)	3.37 ± 0.43	3.55 ± 0.21	3.47 ± 0.27	3.50 ± 0.25	–	–	–	–
Ovaries (mg)	–	–	–	–	79 ± 17N	84 ± 13	81 ± 6	84 ± 12
Thyroid gland (mg)	27 ± 5	31 ± 6	27 ± 8	29 ± 5	21 ± 8	23 ± 5	22 ± 7	26 ± 6
Pituitary (mg)	15 ± 2	15 ± 3	15 ± 2	15 ± 2	15 ± 4	17 ± 3	16 ± 3	16 ± 3
Thymus (mg)	383 ± 62	339 ± 35	331 ± 83	311 ± 90	271 ± 50	253 ± 49	277 ± 53	279 ± 89
Prostate (mg)	1609 ± 245	1533 ± 218	1511 ± 348	1675 ± 171	–	–	–	–
Uterus (mg)	–	–	–	–	469 ± 57N	645 ± 390	590 ± 172	552 ± 138
Seminal vesicles (mg)	1571 ± 159	1609 ± 245	1528 ± 209^c^	1625 ± 96	–	–	–	–
Relative organ weight (%)								
Brain	0.432 ± 0.031	0.419 ± 0.035	0.442 ± 0.055	0.403 ± 0.031	0.790 ± 0.098	0.759 ± 0.078	0.752 ± 0.074	0.743 ± 0.085
Heart	0.289 ± 0.014N	0.294 ± 0.022	0.305 ± 0.033	0.283 ± 0.015	0.327 ± 0.033	0.328 ± 0.023	0.330 ± 0.034	0.335 ± 0.026
Liver	2.636 ± 0.146	2.575 ± 0.160	2.521 ± 0.096	2.756 ± 0.232	2.385 ± 0.129N	2.426 ± 0.184	2.380 ± 0.221	2.407 ± 0.077
Kidneys	0.614 ± 0.037	0.602 ± 0.033	0.593 ± 0.046	0.598 ± 0.046	0.644 ± 0.052	0.659 ± 0.054^b^	0.651 ± 0.052	0.682 ± 0.063
Spleen	0.168 ± 0.020N	0.157 ± 0.027	0.146 ± 0.013	0.145 ± 0.010^d^	0.178 ± 0.029	0.173 ± 0.027	0.173 ± 0.027	0.177 ± 0.018
Adrenal glands	0.012 ± 0.002	0.012 ± 0.002	0.011 ± 0.001	0.011 ± 0.002	0.023 ± 0.002	0.024 ± 0.003	0.022 ± 0.003	0.022 ± 0.003
Testes	0.636 ± 0.070	0.654 ± 0.072	0.663 ± 0.075	0.612 ± 0.039	–	–	–	–
Ovaries	–	–	–	–	0.030 ± 0.005	0.031 ± 0.005	0.030 ± 0.003	0.030 ± 0.003
Thyroid gland	0.005 ± 0.001	0.006 ± 0.001	0.005 ± 0.002	0.005 ± 0.001	0.008 ± 0.003	0.008 ± 0.001	0.008 ± 0.002	0.009 ± 0.002
Pituitary	0.003 ± 0.000	0.003 ± 0.001	0.003 ± 0.000	0.003 ± 0.000	0.006 ± 0.001	0.006 ± 0.001	0.006 ± 0.001	0.005 ± 0.001
Thymus	0.073 ± 0.015	0.063 ± 0.009	0.063 ± 0.014	0.055 ± 0.015^d^	0.103 ± 0.022	0.094 ± 0.019	0.103 ± 0.020	0.098 ± 0.025
Prostate	0.302 ± 0.036	0.282 ± 0.044	0.288 ± 0.064	0.293 ± 0.027	–	–	–	–
Uterus	–	–	–	–	0.179 ± 0.022N	0.233 ± 0.126	0.219 ± 0.068	0.196 ± 0.043
Seminal vesicle	0.296 ± 0.012N	0.295 ± 0.037	0.297 ± 0.035^c^	0.285 ± 0.023	–	–	–	–

Abbreviations: *A. tubingensis*, *Aspergillus tubingensis*; AG, arabinase from nongenetically modified *A. tubingensis* strain GPA41; *N*, nonparametric analysis; TOS, total organic solids.

Values represent the mean ± standard deviation.

a Control animals were administered distilled water.

b The kidneys of 9 animals were analyzed statistically.

c The seminal vesicles of 8 animals were analyzed statistically.

d Significantly different from control (*p* ≤ .05).

### 3.2 Genotoxicity studies

#### 3.2.1 Bacterial reverse mutation test (Ames test)

Increased numbers of revertant colonies in the treatment groups by two times or more relative to the negative control were observed in the preliminary and concentration‐finding studies in *S. typhimurium* TA100 and TA1535 in the absence or presence of S9, as well as in TA1537 in the absence of S9 (data not shown). An increase of greater than twofold in the number of revertant colonies compared to the negative control value also was observed in TA98 in the concentration‐finding study without S9. Accelerated growth in background bacteria was observed in the preliminary study at 15.3 mg TOS/plate in the case of all tester organisms and in the concentration‐finding study, at the three highest concentrations for TA1537 (1.7, 5.1, and 15.3 mg TOS/plate) in the absence of S9 and at the two highest concentrations (5.1 and 15.3 mg TOS/plate) for TA1537 in the presence of S9 and for all other tester organisms (with and without S9).

In the main study conducted with the preincubation method, the number of revertant colonies increased by two times or greater in TA98 at the two highest concentrations tested (7.7 and 15.3 mg TOS/plate) in the presence of S9 relative to the negative control (see Table 4). In *E. coli* WP2*uvrA* in the presence and absence of S9 and in TA1537 in the presence of S9, the number of revertant colonies was comparable to the number of revertant colonies in the negative control. An accelerated growth in background bacteria with cloudy medium was observed in all strains exposed to the three highest concentrations of AG concentrate (3.8, 7.7, and 15.3 mg TOS/plate).

**Table 4:** Results of bacterial reverse mutation test of AG concentrate (main study using preincubation method)

Concentration (mg TOS/plate)	Revertant colonies per plate (mean ± *SD*)					
	*Escherichia coli* WP2*uvrA*		*Salmonella typhimurium*			
			TA98		TA1537	
	‐S9	+S9	‐S9	+S9	‐S9	+S9
Negative control (distilled water)^a^	37 ± 11 (29.8 ± 5.1; 18.7–40.9)^b^	29 ± 4 (28.3 ± 5.9; 16.8–39.7)^b^	− (26.5 ± 4.3; 16.8–36.2)^b^	49 ± 7 (34.5 ± 6.9; 19.1–49.9)^b^	− (10.7 ± 2.9; 3.7–17.7)^b^	19 ± 3 (19.4 ± 6.3; 7.4–31.4)^b^
0.48	–	‐	–	54 ± 0	–	–
0.96	28 ± 4	32 ± 5	–	47 ± 13	–	13 ± 2
1.9	33 ± 4	35 ± 3	–	63 ± 4	–	16 ± 4
3.8	48 ± 6^c^	37 ± 4^c^	–	72 ± 4^c^	–	28 ± 6^c^
7.7	39 ± 2^c^	33 ± 6^c^	–	100 ± 4^c^, ^d^	–	28 ± 7^c^
15.3	27 ± 2^c^	21 ± 7^c^	–	104 ± 13^c^, ^d^	–	36 ± 4^c^
Positive control^e^, ^f^	140 ± 5^d^ (115.1 ± 14.7; 89.4–140.9)^b^	965 27^d^ (744.7 ± 120.9; 510.5–978.9) b	− (695.1 ± 46.2; 571.0–819.2)^b^	425 ± 35^d^ (299.9 ± 90.3; 112.4–487.5)^b^	− (287.3 ± 68.3; 134.7–439.8)^b^	204 ± 15^d^ (139.7 ± 38.1; 72.5–206.9)^b^

Abbreviations: –, not applicable; ‐S9, in the absence of S9; +S9, in the presence of S9; 2‐AA, 2‐aminoanthracene; 9‐AA, 9‐aminoacridine hydrochloride; *A. tubingensis*, *Aspergillus tubingensis*; AF‐2, 2‐(2‐furyl)‐3‐(5‐nitro‐2‐furyl) acrylamide; AG, arabinase from nongenetically modified *A. tubingensis* strain GPA41; *SD*, standard deviation; TOS, total organic solids.

a 100 µl/plate.

b Historical control values for the laboratory (mean ± *SD* and range).

c The growth of background lawn of bacteria was accelerated, and the plates looked turbid.

d Mean number of revertant colonies ≥ 2 times that of the negative control colonies.

e Positive control ‐S9: TA98 = 0.1 µg/plate AF‐2; TA1537 = 80 µg/plate 9‐AA; WP2*uvrA* = 0.01 µg/plate AF‐2.

f Positive control + S9: TA98 = 0.5 µg/plate 2‐AA; TA1537 = 2.0 µg/plate 2‐AA; WP2*uvrA* = 10 µg/plate 2‐AA.

In the main study using the modified (treat‐and‐wash) method, the number of revertant colonies was less than two times that of the negative control in TA100, TA1535, and TA98 in the presence and absence of S9 and in TA1537 in the absence of S9, and the results were replicated in the confirmatory study (Table 5). Neither accelerated growth in background bacteria, nor inhibition of bacterial growth were observed at any concentration in the assays (main and confirmatory) conducted using the treat‐and‐wash method. Growth of bacteria also was not inhibited in the main study using the preincubation method. The positive control in each study resulted in an increase in number of revertant colonies by two times or greater relative to the negative control. No precipitation of the test article was reported in any of the studies at the start of treatment or at the time of colony counting. The results of the negative and positive controls were all within the acceptable range based on the laboratory's historical data, confirming that the study was properly conducted.

**Table 5:** Results of bacterial reverse mutation test of AG concentrate (main and confirmatory study using the “treat‐and‐wash” method)

Concentration (mg TOS/plate)	Revertant colonies per plate (mean ± *SD*)							
	TA100		TA1535		TA98		TA1537	
	‐S9	+S9	−S9	+S9	−S9	+S9^a^	−S9	+S9
Main study								
Negative control (distilled water)^b^	153 ± 16 (128.2 ± 14.4; 91.9–164.6)^c^	172 ± 18 (126.2 ± 12.4; 107.0–145.4)^c^	25 ± 3 (13.4±;2.9; 6.4–20.5)^c^	35 ± 3 (11.4 ± 3.5; 4.7–18.1)^c^	23 ± 5 (26.5 ± 4.3; 16.8–36.2)^c^	27 ± 6 (34.5 ± 6.9; 19.1–49.9)^c^	9 ± 2 (10.7 ± 2.9; 3.7–17.7)^c^	− (19.4 ± 6.3; 7.4–31.4)^c^
0.48	158 ± 12	196 ± 28	33 ± 3	44 ± 6	35 ± 3	22 ± 4	13 ± 4	–
0.96	143 ± 4	192 ± 15	31 ± 3	42 ± 8	31 ± 7	21 ± 2	13 ± 5	–
1.9	159 ± 21	199 ± 17	31 ± 9	35 ± 5	27 ± 4	22 ± 3	14 ± 2	–
3.8	155 ± 20	206 ± 14	36 ± 3	39 ± 8	30 ± 5	27 ± 3	15 ± 3	–
7.7	147 ± 17	244 ± 24	41 ± 7	44 ± 5	32 ± 4	30 ± 2	13 ± 4	–
15.3	126 ± 10	249 ± 8	40 ± 2	62 ± 8	33 ± 5	20 ± 2	9 ± 2	–
Positive control^d^, ^e^	812 ± 82^f^	922 ± 72^f^	51 ± 11^f^	115 ± 19^f^	189 ± 31^f^	179 ± 15^f^	2,067 ± 153^f^	–
Confirmative study								
Negative control (distilled water)^b^	137 ± 9	138 ± 23	7 ± 4	9 ± 2	26 ± 6	16 ± 3	5 ± 3	–
0.96	138 ± 10	161 ± 11	10 ± 4	8 ± 2	28 ± 5	25 ± 4	2 ± 2	–
1.9	139 ± 13	162 ± 13	9 ± 2	12 ± 4	22 ± 5	17 ± 2	7 ± 2	–
3.8	133 ± 8	160 ± 15	8 ± 2	9 ± 3	27 ± 4	23 ± 6	6 ± 2	–
7.7	133 ± 12	177 ± 14	6 ± 4	12 ± 4	26 ± 2	21 ± 5	4 ± 1	–
15.3	127 ± 7	190 ± 9	9 ± 4	14 ± 1	30 ± 1	17 ± 5	6 ± 2	–
Positive control^d^, ^e^	709 ± 122^f^ (696.6 ± 84.7; 488.4–904.8)^c^	344 ± 68^f^ (812.0 ± 200.3; 390.7–1,233.2)^c^	30 ± 5^f^ (667.7 ± 47.9; 519.3–816.1)^c^	176 ± 11^f^ (332.0 ± 54.7; 246.9–417.2)^c^	155 ± 25^f^ (695.1 ± 46.2; 571.0–819.2)^c^	118 ± 14^f^ (299.9 ± 90.3; 112.4–487.5)^c^	1,168 ± 82^f^ (287.3 ± 68.3; 134.7–439.8)^c^	− (139.7 ± 38.1; 72.5–206.9)^c^

Abbreviations: –, not applicable; −S9, in the absence of S9; +S9, in the presence of S9; 2‐AA, 2‐aminoanthracene; 4‐NQO, 4‐nitroquinoline 1‐oxide; 9‐AA, 9‐aminoacridine hydrochloride; *A. tubingensis*, *Aspergillus tubingensis*; AF‐2, 2‐(2‐furyl)‐3‐(5‐nitro‐2‐furyl) acrylamide; AG, arabinase from nongenetically modified *A. tubingensis* strain GPA41; *SD*, standard deviation; TOS, total organic solids.

a TA98 tested positive in the concentration‐finding study in the absence of S9; in the presence of S9, negative results were obtained. Therefore, TA98 with S9 was included in the main study under the conditions of the preincubation method; however, because positive results were obtained in the main preincubation study with S9, TA98 was retested with and without S9 using the treat‐and‐wash method.

b 100 µL/plate.

c Historical control values for the laboratory (mean ± *SD* and range).

d Positive control ‐S9: TA100 and TA98 = 0.1 µg/plate AF‐2; TA1535 = 2.0 µg/plate 4‐NQO; TA1537 = 20 µg/plate 9‐AA.

e Positive control + S9: TA100 = 2.0 µg/plate 2‐AA; TA1535 = 4.0 µg/plate 2‐AA; TA98 = 1.0 µg/plate 2‐AA.

f Mean number of revertant colonies ≥ 2 times that of the negative control colonies.

#### 3.2.2 In vitro mammalian chromosome aberration test

In the short‐term assay, the lowest concentration of AG concentrate to yield a relative growth rate of less than 50% in the absence and presence of S9 was 12.3 and 15.3 mg TOS/mL, respectively, corresponding to a relative growth rate of 41.6 and 41.5%, respectively (see Table 6). Thus, the concentrations that were selected for assessment of chromosomal aberrations were 7.8, 9.8, and 12.3 mg TOS/mL and 9.8, 12.3, and 15.3 mg TOS/mL, respectively. The incidence of structural aberrations was statistically increased at all concentrations in the absence of S9 (*p* ≤ .025) and at a concentration of 12.3 mg TOS/mL or higher in the presence of S9 (*p* ≤ .025). The increases in the incidence of structural chromosomal aberrations observed in the short‐term assay in the absence and presence of S9 occurred in a concentration‐dependent manner (*p* ≤ .025). The incidences of polyploid cells were comparable in all treatment groups in the short‐term assays relative to the negative control. The positive control in the absence and presence of S9 both exhibited high incidences of structural aberrations (*p* ≤ .025).

**Table 6:** In vitro mammalian chromosome aberration test conducted with AG concentrate

Concentration (mg TOS/mL)	Relative cell growth (%)	Number of cells with structural aberrations						Number of cells with aberrations—gap (%)	Number of polyploid cells (%)
		Gap	ctb	cte	csb	cse	Others		
6‐hr short‐term treatment: ‐S9									
Negative control (100 µl/ml saline)	100.0	1	2	0	0	1	0	3 (1.5)^b^ (0.7 ± 1.0; 0.0–3.6)^a^	0 (0.0) (0.1 ± 0.3; 0.0–0.9)^a^
7.8	90.2	0	4	11	16	5	0	25 (12.5)^c^	0 (0.0)
9.8	56.5	0	5	23	45	15	0	63 (31.5)^c^	3 (1.5)
12.3	41.6	0	12	44	36	10	0	71 (35.5)^c^	1 (0.5)
15.3	30.3	–	–	–	–	–	–	–	–
Positive control (0.1 µl/ml MMC)	62.7	7	49	92	1	0	0	120 (60.0)^c^ (59.7 ± 9.2; 32.2–87.2)^a^	1 (0.5)
6‐hr short‐term treatment: +S9									
Negative control (100 µl/ml saline)	100.0	0	0	0	0	0	0	0 (0.0)^b^ (0.2 ± 0.4; 0.0–1.3)^a^	0 (0.0) (0.2 ± 0.4; 0.0–1.3)^a^
9.8	100.2	2	2	1	1	0	0	3 (1.5)	0 (0.0)
12.3	69.3	1	1	5	1	0	0	6 (3.0)^c^	
15.3	41.5	3	9	13	0	0	0	17 (8.5)^c^	0 (0.0)
Positive control (12.5 µl/ml CP)	86.3	5	54	126	1	0	0	137 (68.5)^c^ (41.5 ± 9.4; 13.4–69.5)^a^	0 (0.0)
24‐hr continuous treatment									
Negative control (100 µl/ml saline)	100.0	0	0	0	0	0	0	0 (0.0) (0.6 ± 0.9; 0.0–3.3)^a^	0 (0.0) (0.1 ± 0.3; 0.0–1.1)^a^
0.66	132.9	1	0	1	0	0	0	1 (0.5)	0 (0.0)
1.1	138.5	0	0	1	0	0	0	1 (0.5)	0 (0.0)
1.8	127.0	3	0	1	0	0	0	1 (0.5)	0 (0.0)
3.1	105.1	–	–	–	–	–	–	–	–
Positive control (0.05 µl/ml MMC)	96.0	0	43	88	0	0	0	111 (55.5)^c^ (43.4 ± 9.9; 13.8–73.1)^a^	0 (0.0)

Abbreviations: ‐, not applicable; ‐S9, in the absence of S9; +S9, in the presence of S9; *A. tubingensis*, *Aspergillus tubingensis*; AG, arabinase from nongenetically modified *A. tubingensis* strain GPA41; CP, cyclophosphamide; MMC, mitomycin C; TOS, total organic solids.

a Historical control values for the laboratory (mean ± *SD* and range).

b Significant correlation with dosage levels (Cochran–Armitage trend test): *p* ≤ .025.

c Significant difference from control (Fisher's exact test): *p* ≤ .025.

In the initial continuous assay conducted with AG concentrate, concentrations in the range of 5.0 to 15.3 mg TOS/mL, a significant portion of the mitotic cells displayed c‐mitosis (mitosis with disturbed spindle function) at all tested concentrations, such that almost no cells with analyzable mitosis were available, and therefore, microscopic examination for chromosomal aberrations was not conducted (data not shown). In the additional continuous treatment test conducted with lower concentrations of AG concentrate (0.24 to 5.1 mg TOS/mL), no growth inhibition was observed at any of the tested concentrations; however, evaluation of chromosomal aberrations was impossible at concentrations of 3.1 mg TOS/mL and greater due to the frequent presence of c‐mitosis figures, and thus, only concentrations of ≤ 1.8 mg TOS/mL were examined microscopically for chromosomal aberrations. As shown in Table 6, at the concentrations selected for examination of chromosomal aberrations, incidences of chromosomal aberrations and polyploid cells following treatment with AG concentrate were all comparable to those in the negative control group. Cells treated with positive control exhibited high incidence of structural aberrations (*p* ≤ .025). Both the negative and positive control groups had results within the ranges of the laboratory's historical data indicating the validity of the performed study. Precipitation of the test article was not observed in any of the assays.

The D_20_ value for AG concentrate was calculated to be 50.2 mg/mL; the TR value was determined to be 0.264. The laboratory's D_20_ and TR values for the positive control, mitomycin C, were 3.2 × 10^‐5^ mg/mL and 10^6^, respectively; for cyclophosphamide, the values were 9.3 × 10^‐3^ mg/mL and 4.2 × 10^3^, respectively.

#### 3.2.3 In vivo mammalian erythrocyte micronucleus test

No significant increases were noted in the frequency of MNIE in any of the groups of rats administered AG concentrate at doses of up to 1,530 mg TOS/kg body weight compared to the negative control (Table 7). The ratio of IE to total analyzed erythrocytes (i.e., as a marker of bone marrow proliferation) was calculated to be 71.8, 70.0, 70.8, and 70.7% in the control, low‐, mid‐, and high‐dose groups, respectively, with no significant decreases identified between any of the test group and negative control group values. In contrast, a marked and statistically significant increase (*p* ≤ .025) was observed in the frequency of MNIE in the positive control group compared to the negative control group (Table 7). The ratio of IE to the total number of erythrocytes analyzed was slightly reduced in the positive control group relative to the negative control. The frequency of MNIE and the ratio of IE to analyzed erythrocytes in the negative and positive control groups were within the range of the laboratory's historical data, supporting the validity of this study. Body weight gain and general condition of the animals (data not shown) were normal in all groups of animals throughout the entire study.

**Table 7:** Results of the in vivo mammalian erythrocyte micronucleus test in male rats orally (gavage) administered AG concentrate once daily for 2 days

Dose (mg TOS/kg bw)	Number of animals	Frequency of MNIE [%]^a^ (mean ± *SD*)	Range of MNIE/2000 IE (min‐max)	Ratio of IE [%]^b^ (mean ± *SD*)
Negative control (10 ml/kg bw distilled water)	5	0.15 ± 0.09 (0.16 ± 0.10; 0.00–0.46)^c^	1–6	71.8 ± 1.6 (56.2 ± 9.3; 28.3–84.1)^c^
383	5	0.15 ± 0.10	1–6	70.0 ± 4.7
767	5	0.22 ± 0.12	2–7	70.8 ± 2.8
1,530	5	0.11 ± 0.07	0–4	70.7 ± 6.5
Positive control (10 mg/kg bw CP)	5	2.14 ± 0.44^d^ (2.92 ± 0.90; 0.22–5.62)^c^	31–52	68.1 ± 3.2 (50.6 ± 6.0; 32.6–68.6)^c^

Abbreviations: *A. tubingensis*, *Aspergillus tubingensis*; AG, arabinase from nongenetically modified *A. tubingensis* strain GPA41; bw, body weight; CP, cyclophosphamide; IE, immature erythrocyte; MNIE, micronucleated immature erythrocyte; *SD*, standard deviation; TOS, total organic solids.

a Calculated as [number of MNIE]/ [number of IE analyzed] x 100.

b Calculated as [number of IE]/ [number of total erythrocytes analyzed] x 100.

c Historical control values for the laboratory (mean ± *SD* and range).

d Significantly different from negative control (Kastenbaum and Bowman method, *p* ≤ .025).

#### 3.2.4 In vivo comet test

Macroscopic examinations of the glandular stomach and duodenum were unremarkable in all groups of animals treated with AG concentrate (data not shown). AG concentrate‐treated animals also were observed to gain weight, and no clinical signs of toxicity were noted (data not shown). No statistically significant increases in the % tail DNA were observed in stomach (2.94, 3.17, and 3.16) or duodenum (1.71, 1.57, and 1.89) cells of AG‐treated groups at any dose relative to the negative control group (stomach cells: 3.48 and duodenum cells: 1.76) (Table 8). Administration of AG concentrate also was not associated with any apparent increases in the frequency of hedgehogs in either cell type (as compared to the negative control group values) (Table 8). In contrast, statistically significant increases in % tail DNA were observed in both stomach and duodenum cells in the positive control group, supporting the validity of the study. Furthermore, the % tail DNA in the negative control group was within the acceptable ranges calculated based on the laboratory's historical data, which also confirmed the validity of the study.

**Table 8:** Results of the in vivo mammalian alkaline comet assay conducted in male rats orally (gavage) administered AG concentrate once daily for 2 days

Substance Dose (mg TOS/kg bw)	Number of animals	Glandular stomach		Duodenum	
		% tail DNA^a^ (mean ± *SD*)	Frequency of hedgehogs (%) (mean ± *SD*)	% tail DNA^a^ (mean ± *SD*)	Frequency of hedgehogs (%) (mean ± *SD*)
Negative control (distilled water; 10 ml/kg bw)	5	3.48 ± 0.46 (2.80 ± 1.84; 0.00–8.32)^b^	2.0 ± 1.1	1.76 ± 0.29 (1.31 ± 0.90; 0.00–4.01)^b^	2.9 ± 2.2
383	5	2.94 ± 0.35	2.0 ± 1.1	1.71 ± 0.33	2.5 ± 1.6
765	5	3.17 ± 0.39	1.3 ± 1.2	1.57 ± 0.25	4.0 ± 2.1
1,530	5	3.16 ± 0.62	1.2 ± 0.6	1.89 ± 0.35	3.9 ± 1.6
Positive control (EMS; 200 mg/kg bw)	5	31.29 ± 3.42	3.6 ± 1.5^c^	28.65 ± 3.42	2.9 ± 0.8^c^

Abbreviations: *A. tubingensis*, *Aspergillus tubingensis*; AG, arabinase from nongenetically modified *A. tubingensis* strain GPA41; bw, body weight; EMS, ethyl methanesulfonate; *SD*, standard deviation; TOS, total organic solids.

a The mean % tail DNA in each group was calculated from that in each animal based on the median % tail DNA in each slide.

b Historical control values for the laboratory (mean ± *SD* and range).

c Significantly different from negative control (Aspin–Welch's *t* test, *p* ≤ .05).

## 4 DISCUSSION

AG, arabinase from nongenetically modified *A. tubingensis* strain GPA41, has potential commercial applications in the food industry, particularly in the processing of fruit juices. In fruit juice processing, arabinase acts to clarify juice by degrading arabinose polymers (arabinan), the presence of which is associated with juice cloudiness or haze. The safety of AG for use in food processing for human consumption was evaluated in this study by means of a battery of toxicological testing, consisting of a 90‐day repeated oral dose toxicity test conducted in rats and a series of in vitro and in vivo genotoxicity tests, including a bacterial reverse mutation test, an in vitro mammalian chromosomal aberration test, an in vivo mammalian erythrocyte micronucleus test, and an in vivo comet assay.

To evaluate potential toxicity following repeated administration, AG concentrate was administered to rats by oral gavage at doses of up to 1,530 mg TOS/kg body weight/day for 3 months. No mortality and no test substance‐related symptoms of toxicity or ophthalmological findings were observed. Food consumption, body weight, and body weight gain did not differ statistically between control and test group animals. Clinical chemistry revealed statistically significant increases in levels of potassium in high‐dose males; however, the change in plasma potassium was not accompanied by any significant variability in urine potassium, or variations in other electrolytes and individual animal values were all within the laboratory's historical data (data not provided). Furthermore, a similar change was not observed in treated females. As such, the difference was considered as not related to the administration of the enzyme. The decreases in relative thymus and spleen weights of high‐dose males may have been the result of slightly greater (albeit not statistically significant) body weights at the end of dosing in high‐dose animals compared to controls. Similarly, the increase in absolute kidney weights in high‐dose females was also attributed to the slight end‐of‐study body weight differences between test and control animals. The gross abnormalities observed in the spleen, lungs, liver, and kidneys in males, and stomach and ileum in females were judged to be spontaneous occurrences based on the incidence, severity, and morphological characteristics of the findings. Likewise, the histological variabilities of the glandular stomach and pancreas in the high‐dose animals were slight focal changes, with the severity, and morphological characteristics also consistent with those typically seen for this strain and age of rats. Therefore, the microscopic lesions also were determined to be spontaneous findings. Histological examination of the Zymbal's gland (auditory sebaceous gland) confirmed the nodule identified in one high‐dose female at gross necropsy as adenoma. Although not considered a common spontaneous lesion in rats, incidences of Zymbal's gland tumors in control animals of toxicological studies have been identified (Dinse, Peddada, Harris, & Elmore, 2010; Rudmann et al., 2012; Weber, 2017). Zymbal's gland adenoma also had been previously identified as an isolated finding in other repeated rat oral dose toxicity studies conducted at the laboratory (data not provided). Therefore, the single occurrence of adenoma of the Zymbal's gland in only one female rat in this study also was considered a spontaneous occurrence. Based on the results of this study, it was concluded that AG concentrate was not associated with any adverse effects at a dose of up to 1,530 mg TOS/kg body weight/day, the highest dose tested. The study therefore supports the highest dose tested (1,530 mg TOS/kg body weight/day) as the no‐observed‐adverse‐effect level for AG concentrate in rats.

Following exposure to AG concentrate, significant increases in revertant colonies (twofold or greater) were observed in *S*. *typhimurium* strains TA100 and TA1535 in the presence and absence of metabolic activation and in TA98 and TA1537 in the absence of metabolic activation when tested in initial preliminary and dose‐finding bacterial reverse mutation studies under the conditions of the preincubation method. A similar increase in the number of revertants also was observed in the main study with TA98 tested with metabolic activation under preincubation conditions. The responses observed in the Ames assay under the preincubation conditions were attributed to the release of amino acids (e.g., histidine) into the culture medium, which is commonly observed with proteinaceous materials and is recognized as a confounding factor in bacterial reverse mutation tests involving enzymes (EFSA, 2014; Thompson, Morley, Kirkland, & Proudlock, 2005). Free amino acids released into the culture medium can enhance the growth of the test bacteria leading to additional spontaneous mutations (EFSA, 2014; Thompson et al., 2005). Increases in spontaneous mutations resulting from the presence of free amino acids in the medium can be misinterpreted as a substance‐related genotoxic response when in fact it is the amino acid content of the culture medium that is responsible for the overall increase in the bacterial growth and greater potential for mutations to occur. Additionally, amino acids released during the incubation can be associated with overgrowth of nonrevertant background lawn bacteria, which also limits the interpretability of the results (EFSA, 2014; Thompson et al., 2005). In such instances, the treat‐and‐wash protocol in which bacteria are washed after treatment with the test article to remove free amino acids is considered as an acceptable modification of the preincubation method (EFSA, 2014; Thompson et al., 2005). Similar increases in the number of revertants, which also were considered as likely due to the copresence of free amino acids, were reported in another Ames assay with a different enzyme preparation (enzymes nuclease P1 from *Penicillium citrium*) when testing was conducted under preincubation conditions (Okado et al., 2016). When testing was repeated using the treat‐and‐wash method, an increase in revertant colonies was not observed in any of the tester strains, and thus, *P. citrium*‐derived nuclease P1 was concluded to be nonmutagenic.

For tester organism in which positive responses were observed under the conditions of the preincubation method, the assay was repeated using the modified (treat‐and‐wash) method, to eliminate any effects resulting from the presence of free amino acids. Under the conditions of the treat‐and‐wash method, the number of revertant colonies was consistently less than two times that of the negative control in all tested strains of *S. typhimurium* following exposure to AG concentrate in both the main treat‐and‐wash assay, and the follow‐up assay, which was conducted to confirm reproducibility of the results (the “confirmatory” assay). The absence of positive responses obtained with the treat‐and‐wash method confirmed that the positive findings observed using the standard preincubation method were a result of the presence of free amino acids in the culture medium. Accelerated background lawn (bacterial growth), which had been observed under the conditions of the preincubation assay, was not observed with the treat‐and‐wash method and was therefore also considered a result of the presence of free amino acids in the culture medium. Given the nature of the test material, the treat‐and‐wash method can be considered more appropriate for the testing of the mutagenic potential of the AG concentrate than the standard preincubation protocol. On the basis of the study using the modified method, AG was considered to lack mutagenic potential in the in vitro bacterial reverse mutation test and the increases in the number of revertant colonies observed in the assay using the standard method were attributed to the presence of free amino acids.

The in vitro chromosomal aberration test was used to evaluate the clastogenic potential of AG in cultured CHL fibroblast cells. Under the conditions of the “short‐term treatment” consisting of a 6‐hr exposure period to the test article, followed by an 18‐hr expression period, statistically significant and concentration‐dependent increases were observed in the incidence of chromosome aberrations at all concentrations selected for examination of chromosome aberrations without metabolic activation (7.8 to 12.3 mg TOS/mL). In the presence of metabolic activation, the number of cells with aberrations also increased in a concentration‐dependent manner; however, statistical significance was attained only at concentrations of 12.3 mg TOS/mL and greater, indicating that the increases in the number of cells with aberrations were diminished in the presence of metabolic activation.

Tested under the continuous assay conditions at concentrations similar to those used in the short‐term exposure assays, the number of analyzable cells was severely limited by the presence of c‐mitosis figures at all tested concentrations. As such, testing was repeated at lower concentrations (0.24 to 5.1 mg TOS/mL). Although evaluation of cells for chromosomal aberrations was again impeded by significant incidences of c‐mitosis at the two highest tested concentrations of AG concentrate (3.1 and 5.1 mg TOS/mL), at concentrations of up to 1.8 mg TOS/mL, no significant effects on the incidence of chromosome aberrations, incidence of polyploid cells, or rate of cell growth were observed in comparison with the negative control. Based on the results of the study, it was concluded that AG induces structural chromosome aberrations in CHL cell lines as observed under the conditions of the short‐term treatment assay; however, in comparison with known clastogens, including the positive controls used in this study (mitomycin C and cyclophosphamide), the clastogenic activity of AG concentrate, as determined on the basis of the D_20_ and the TR values, was considered to be low. As such, the results of the assay indicate a weakly clastogenic response.

Based upon the positive results obtained in the in vitro chromosome aberration study, follow‐up studies including a mammalian micronucleus assay and a comet assay were conducted in vivo to further investigate the potential genotoxicity of AG. Following treatment of male Crl:CD (*SD*) [SPF] rats with AG concentrate at doses of up to 1,530 mg TOS/kg body weight/day for two consecutive days, there were no statistically significant increases in the frequency of MNIE in any of the treatment groups, when compared to the negative control group. Additionally, the ratio of IE showed no statistically significant decrease among the treated animals. Therefore, under the conditions of this study it is concluded that AG did not induce micronucleated erythrocytes in rat bone marrow cells.

In order to further examine the difference between the positive results of the in vitro genotoxicity assay and the negative results in the in vivo micronucleus assay, the possibility of DNA damage following oral exposure to AG was also assessed in rats in an alkaline comet assay. In conducting the comet assay, consideration was given to the possibility that the absence of effects in the in vivo micronucleus assay could possibly be a result of a lack of systemic exposure of the bone marrow cells to intact enzyme, which would be expected to be subjected to preabsorptive breakdown of the protein matter in the gastrointestinal tract. It was also recognized that the in vivo micronucleus assay that involved use of bone marrow cells did not address potential site‐of‐contact genotoxicity concerns of viable enzyme on rapidly dividing cells in the mucosa of the stomach and upper small intestine. For highly reactive substances that are not systemically available, site‐of‐contact tissues may be more appropriate for evaluation of a possible genotoxic effect particularly if there is no kinetic evidence that the agent reaches any postabsorptive tissue suitable for genotoxicity testing (EFSA, 2011; OECD, 2016). Ensuring therefore that the sampled tissue has in fact received adequate exposure to the test article is an important consideration in the testing of chemicals for genotoxicity potential (Vasquez, 2012). Accordingly, preabsorptive cells of the stomach and duodenum that would be expected to come into direct contact with the test article immediately following ingestion were selected for the testing of AG in the comet assay, particularly since no evidence of toxicity to the target organ (bone marrow cells) was observed in the in vivo micronucleus assay (i.e., no differences in the IE ratio between test and negative control animals). Oral administration of AG concentrate to rats at doses of up to 1,530 mg TOS/kg body weight on two consecutive occasions (21‐hr interval between dosing) was not associated with any significant changes in the % tail DNA or hedgehog frequency in stomach or duodenum cells. Macroscopic examination of both organs (stomach and duodenum) also was unremarkable; since DNA damage was not observed in the cells of the sampled organs, histopathology was not conducted. No adverse effects and normal body weight gain were reported in both in vivo genotoxicity studies. On the basis of the results of the in vivo mammalian micronucleus and alkaline comet assay, AG is considered to lack any genotoxic potential, which includes both stomach and duodenum as potential target organs.

Assessment of the safety of an enzyme for use in food must also consider the source organism (Pariza & Johnson, 2001). Not only are the properties of an enzyme affected by the microorganism from which it is derived (e.g., optimal pH and temperature conditions, enzymatic activity) (Beldman, Searle‐van Leeuwen, Siliha, & Voragen, 1993), but the safety of the source organism per se, such as its toxigenic and pathogenic potential, also should be considered in an overall assessment of the enzyme's safety (Pariza & Johnson, 2001). Although no other toxicological studies appear to have been conducted with arabinase from *A. tubingensis*, a study was identified in the published literature in which the safety of another *A. tubingensis*‐derived enzyme (glucose oxidase) was assessed (Kriaa et al., 2015). In this study, no adverse effects were associated with the administration of a heat‐inactivated glucose oxidase (enzyme activity not reported) preparation sourced from *A. tubingensis* strain CTM 507 to male Wistar rats (8 animals/group) via gavage at 0.4 mg/kg body weight/day for a period of 4 weeks. While concern has been previously raised regarding the potential of *A. tubingensis* to produce secondary metabolites of unknown toxicities (EFSA, 2009a), the results of the studies described herein, as well as the study by Kriaa et al. (2015), support the absence of any substances associated with adverse effects in the enzyme preparations, suggesting that *A. tubingensis* does not produce toxic metabolites. Furthermore, *A. tubingensis* GPA41, the production strain used as the source of AG, tested negative when screened for the production of a series of common mycotoxins, including ochratoxin A and fumonisin B1 and B2, and other less common secondary metabolites also possibly produced by *A. tubingensis*, including nigragillins, malformins, and naptho‐γ‐pyrones (data not shown). Although prior use of *A. tubingensis* as a source organism for enzymes intended for use in food processing does not appear to be well documented, it may be anticipated that given the issues with the taxonomic distinction between *A. tubingensis* and *A. niger* species, and likely misclassification of some *A. tubingensis* species as *A. niger*, some enzymes already used in food are in fact products of *A. tubingensis*. Furthermore, despite the somewhat limited indication of prior use in the production of food enzymes, *A. tubingensis* is the gene‐donor organism in the production of transgenic pectin esterase (expressed in *Trichoderma reesei*), which is already Generally Recognized as Safe (GRAS) for use in fruit and vegetable processing, wine, coffee and flavoring production, and grain treatment in the United States (AB Enzymes, 2014).

Overall, the history of commercial use of arabinase in fruit juice processing and other food and beverage applications, combined with the results of the toxicological studies described herein, including the absence of any adverse effects in a repeated oral dose toxicity study in which rats were administered AG concentrate at up to 1,530 mg TOS/kg body weight/day and lack of findings consistent with a genotoxic potential, and the apparent safety of the production organism, collectively suggests the use of AG in food for human consumption to be acceptable.

## CONFLICT OF INTEREST

The authors of this publication have declared that they have no conflicts of interest.

## ETHICAL APPROVAL

This study involved animal testing and followed the Act on Welfare and Management of Animals (Japanese Ministry of the Environment Act No. 105, October 1, 1973, Act No. 79, September 5, 2012, Act No. 38, June 12, 2013, and Act No. 46, May 30, 2014), Standards Relating to the Care and Management of Laboratory Animals and Relief of Pain (Japanese Ministry of the Environment Notification No. 88, April 28, 2006 and Notification No. 84, August 30, 2013), and the testing facility's (Biosafety Research Center—BSRC) Guidelines for Animal Experimentation (October 21, 2013 and February 19, 2014). The protocol and procedures employed were ethically reviewed, and the study was approved by the BSRC Experimental Animal Ethics Committee (Authorization No. 13‐0312A) and by the BSRC Institutional Animal Care and Use Committee (IACUC) (Authorization Nos. 13‐0348A and 16‐0224A).
