# Supplementary material for: Safety evaluation of arabinase (arabinan endo‐1,5‐α‐L‐arabinanase) from Aspergillus tubingensis
Source: Food Sci Nutr. 2019 Dec 17;8(1):456–78. doi: 10.1002/fsn3.1329 (PMC6977434; doi:10.1002/fsn3.1329)
Supplement: Supplementary file 1 [file FSN3-8-456-s001.docx]

## Appendix A: Supplementary Tables

**Supplementary Table 1. Stability of dose formulations prepared with AG concentrate for administration to rats in the 13-week toxicological study**

| **Dose formulation (mg TOS/kg bw)** | **Target enzyme activity (U/mL)** | **Storage timepoint** | | |
| --- | --- | --- | --- | --- |
|  |  | **Baseline (“Just after preparation”)** | **4 Hours**  **(stored on ice)** | **9 Days**  **(Stored in a refrigerator for 8 days and then at room temperature for 24 hours)** |
| 15.3 | 6.9 | 6.180 | 6.331 | 6.606 |
| 1,530 | 690 | 642.0 | 650.4 | 657.3 |
| *A. tubingensis*, *Aspergillus tubingensis*; AG, arabinase from non-genetically modified *A. tubingensis* strain GPA41; TOS, total organic solids. | | | | |

**Supplementary Table 2. Body weight values (mean ± SD) for male and female rats administered AG concentrate by gavage for 13 weeks**

| **Study Day** | **Dose group (mg TOS/kg body weight)** | | | | | | | |
| --- | --- | --- | --- | --- | --- | --- | --- | --- |
|  | **Males (n=10)** | | | | **Females (n=10)** | | | |
|  | **0 (control) ^†^** | **15.3** | **153** | **1,530** | **0 (control) ^†^** | **15.3** | **153** | **1,530** |
| 1 | 165±7 | 165±6 | 165±7 | 164±8 | 136±7 | 136±7 | 136±6 | 136±7 |
| 8 | 226±13 | 225±9 | 223±16 | 228±13 | 163±12 | 163±12 | 164±8 | 166±10 |
| 15 | 280±22 | 282±12 | 277±22 | 287±19 | 181±14 | 179±15 | 184±12 | 185±13 |
| 22 | 333±30 | 341±17 | 332±27 | 348±26 | 200±18 | 196±18 | 204±17 | 204±15 |
| 29 | 375±36 | 385±22 | 374±35 | 396±33 | 217±22 | 215±21 | 219±18 | 223±21 |
| 36 | 415±42 | 423±27 | 410±40 | 438±36 | 229±22 | 226±19 | 236±23 | 241±22 |
| 43 | 448±47 | 454±31 | 438±45 | 472±39 | 242±25 | 239±22 | 248±25 | 252±27 |
| 50 | 472±46 | 479±33 | 461±50 | 499±40 | 251±27 | 250±24 | 256±24 | 263±30 |
| 57 | 495±51 | 504±34 | 478±50 | 526±42 | 258±31 | 259±24 | 266±27 | 271±31 |
| 64 | 514±52 | 522±38 | 498±51 | 547±44 | 267±32 | 270±25 | 274±27 | 281±33 |
| 71 | 531±54 | 542±39 | 514±52 | 565±45 | 273±32 | 274±23 | 279±30 | 286±35 |
| 78 | 545±56 | 558±44 | 534±51 | 582±47 | 277±33 | 282±26 | 285±30 | 293±40 |
| 85 | 558±58 | 571±44 | 550±52 | 597±48 | 282±36 | 287±25 | 289±33 | 298±39 |
| 90 | 557±57 | 572±44 | 551±52 | 596±50 | 281±34 | 285±26 | 288±31 | 296±41 |
| *A. tubingensis*, *Aspergillus tubingensis*; AG, arabinase from non-genetically modified *A. tubingensis* strain GPA41; SD, standard deviation; TOS, total organic solids.  ^†^ Control animals were administered distilled water. | | | | | | | | |

| **Supplementary Table 3. Histopathological findings for male and female rats administered AG concentrate by gavage for 13 weeks** | | | | | | | | |
| --- | --- | --- | --- | --- | --- | --- | --- | --- |
| **Organ or Tissue Observed (Number of animals necropsied)** | **Dose group (mg TOS/kg body weight)** | | | | | | | |
|  | **Males (n=10)** | | | | **Females (n=10)** | | | |
|  | **0 (control) ^†^** | **15.3** | **153** | **1,530** | **0 (control) ^†^** | **15.3** | **153** | **1,530** |
| **Cardiovascular system** | | | | | | | | |
| *Heart* | (10) | (0) | (0) | (10) | (10) | (0) | (0) | (10) |
| Cardiomyopathy | 5 | - | - | 5 | 0 | - | - | 1 |
| **Hematopoietic system** | | | | | | | | |
| *Spleen* | (10) | (0) | (0) | (10) | (10) | (0) | (0) | (10) |
| Cyst | 0 | - | - | 1 | 0 | - | - | 0 |
| *Lymph nodes* | (10) | (0) | (0) | (10) | (10) | (0) | (0) | (10) |
| Infiltration, inflammation cell | 0 | - | - | 0 | 0 | - | - | 1 |
| Inflammatory change, focal | 0 | - | - | 1 | 0 | - | - | 0 |
| Hyperplasia, follicle | 1 | - | - | 0 | 0 | - | - | 0 |
| *Thymus* | (10) | (0) | (0) | (10) | (10) | (0) | (0) | (10) |
| Kuersteiner’s duct/cyst | 1 | - | - | 2 | 3 | - | - | 5 |
| **Respiratory system** | | | | | | | | |
| *Lungs* | (10) | (0) | (0) | (10) | (10) | (0) | (0) | (10) |
| Metaplasia, osseous | 0 | - | - | 2 | 2 | - | - | 1 |
| Aggregation, macrophage | 2 | - | - | 4 | 2 | - | - | 3 |
| Infiltration, inflammatory cell | 0 | - | - | 1 | 0 | - | - | 0 |
| Inflammatory change, focal | 1 | - | - | 3 | 0 | - | - | 0 |
| *Trachea* | (10) | (0) | (0) | (10) | (10) | (0) | (0) | (10) |
| Cyst | 1 | - | - | 1 | 0 | - | - | 0 |
| **Digestive system** | | | | | | | | |
| *Forestomach* | (10) | (0) | (0) | (10) | (10) | (0) | (0) | (10) |
| Cyst | 0 | - | - | 0 | 0 | - | - | 1 |
| *Glandular* *stomach* | (10) | (0) | (0) | (10) | (10) | (0) | (0) | (10) |
| Edema | 0 | - | - | 2 | 0 | - | - | 2 |
| Cyst | 1 | - | - | 0 | 0 | - | - | 0 |
| Single cell necrosis | 0 | - | - | 0 | 0 | - | - | 1 |
| Infiltration, inflammatory cell | 0 | - | - | 2 | 0 | - | - | 0 |
| *Pancreas* (*exocrine*) | (10) | (0) | (0) | (10) | (10) | (0) | (0) | (10) |
| Deposit, pigment | 1 | - | - | 2 | 0 | - | - | 0 |
| Increase in apoptosis | 2 | - | - | 0 | 1 | - | - | 0 |
| Infiltration, inflammatory cell | 1 | - | - | 1 | 1 | - | - | 4 |
| Fatty infiltration | 3 | - | - | 3 | 2 | - | - | 0 |
| Regeneration | 0 | - | - | 2 | 0 | - | - | 1 |
| *Ileum* | (10) | (0) | (0) | (10) | (10) | (0) | (0) | (10) |
| Diverticulum | 0 | - | - | 0 | 0 | - | - | 1 |
| *Colon* | (10) | (0) | (0) | (10) | (10) | (0) | (0) | (10) |
| Hyperplasia, lymphoid tissue | 2 | - | - | 0 | 0 | - | - | 0 |
| *Rectum* | (10) | (0) | (0) | (10) | (10) | (0) | (0) | (10) |
| Edema | 0 | - | - | 0 | 0 | - | - | 2 |
| Hyperplasia, lymphoid tissue | 1 | - | - | 0 | 2 | - | - | 1 |
| *Liver* | (10) | (0) | (0) | (10) | (10) | (0) | (0) | (10) |
| Dilatation, sinusoid, focal | 0 | - | - | 1 | 0 | - | - | 0 |
| Fatty change, hepatocyte | 1 | - | - | 3 | 2 | - | - | 1 |
| Infiltration, inflammatory cell | 10 | - | - | 9 | 9 | - | - | 8 |
| Perivasculitis | 1 | - | - | 1 | 0 | - | - | 0 |
| Extramedullary hematopoiesis | 0 | - | - | 3 | 1 | - | - | 0 |
| *Salivary glands* | (10) | (0) | (0) | (10) | (10) | (0) | (0) | (10) |
| Metaplasia | 0 | - | - | 1 | 0 | - | - | 0 |
| Infiltration, inflammatory cell | 1 | - | - | 0 | 0 | - | - | 0 |
| **Urinary system** | | | | | | | | |
| *Kidneys* | (10) | (0) | (0) | (10) | (10) | (0) | (0) | (10) |
| Cast, hyaline | 0 | - | - | 1 | 1 | - | - | 0 |
| Cyst | 2 | - | - | 1 | 0 | - | - | 0 |
| Dilatation, tubule | 1 | - | - | 0 | 0 | - | - | 0 |
| Hyaline droplet | 5 | - | - | 5 | 0 | - | - | 0 |
| Mineralization | 1 | - | - | 1 | 0 | - | - | 2 |
| Regeneration, tubule | 10 | - | - | 8 | 2 | - | - | 2 |
| Infiltration, inflammatory cell | 3 | - | - | 2 | 1 | - | - | 1 |
| Inflammatory change, focal | 2 | - | - | 3 | 4 | - | - | 2 |
| Dilatation, renal pelvis | 0 | - | - | 1 | 0 | - | - | 0 |
| *Urinary bladder* | (10) | (0) | (0) | (10) | (10) | (0) | (0) | (10) |
| Infiltration, inflammatory cell | 1 | - | - | 0 | 0 | - | - | 0 |
| **Reproductive system** | | | | | | | | |
| *Testes* | (10) | (0) | (0) | (10) | ( - ) | ( - ) | ( - ) | ( - ) |
| Atrophy, seminiferous tubule | 2 | - | - | 0 | - | - | - | - |
| Single cell necrosis | 1 | - | - | 0 | - | - | - | - |
| Vacuolation, seminiferous tubule | 1 | - | - | 0 | - | - | - | - |
| *Epididymides* | (10) | (0) | (0) | (10) | ( - ) | ( - ) | ( - ) | ( - ) |
| Cell debris, lumen | 1 | - | - | 0 | - | - | - | - |
| Deposit, pigment | 0 | - | - | 1 | - | - | - | - |
| Single cell necrosis | 1 | - | - | 0 | - | - | - | - |
| Infiltration, inflammatory cell | 0 | - | - | 1 | - | - | - | - |
| Decrease, sperm | 1 | - | - | 0 | - | - | - | - |
| *Prostate* | (10) | (0) | (0) | (10) | ( - ) | ( - ) | ( - ) | ( - ) |
| Infiltration, inflammatory cell | 6 | - | - | 7 | - | - | - | - |
| Inflammatory change, focal | 1 | - | - | 0 | - | - | - | - |
| *Oviducts* | ( - ) | ( - ) | ( - ) | ( - ) | (10) | (0) | (0) | (10) |
| Hyperplasia, mesothelial | - | - | - | - | 8 | - | - | 7 |
| *Vagina* | ( - ) | ( - ) | ( - ) | ( - ) | (10) | (0) | (0) | (10) |
| Cyst | - | - | - | - | 0 | - | - | 1 |
| **Endocrine system** | | | | | | | | |
| *Pituitary* *gland* | (10) | (0) | (0) | (10) | (10) | (0) | (0) | (10) |
| Cyst | 2 | - | - | 4 | 0 | - | - | 0 |
| Craniopharyngeal derivatives | 1 | - | - | 0 | 0 | - | - | 0 |
| *Thyroid glands* | (10) | (0) | (0) | (10) | (10) | (0) | (0) | (10) |
| Infiltration, inflammatory cell | 1 | - | - | 0 | 1 | - | - | 0 |
| Ectopic thymus | 1 | - | - | 1 | 0 | - | - | 1 |
| Ultimobranchial remnant | 2 | - | - | 4 | 3 | - | - | 2 |
| *Parathyroid glands* | (10) | (0) | (0) | (10) | (10) | (0) | (0) | (10) |
| Hypertrophy | 1 | - | - | 0 | 0 | - | - | 0 |
| *Pancreas (endocrine)* | (10) | (0) | (0) | (10) | (10) | (0) | (0) | (10) |
| Fibrosis | 1 | - | - | 2 | 0 | - | - | 0 |
| **Special sense system** | | | | | | | | |
| *Eyes* | (10) | (0) | (0) | (10) | (10) | (0) | (0) | (10) |
| Atrophy, retina | 1 | - | - | 1 | 0 | - | - | 0 |
| Mineralization | 0 | - | - | 1 | 0 | - | - | 0 |
| Disarrangement | 1 | - | - | 2 | 0 | - | - | 2 |
| Persistent hyaloid artery | 2 | - | - | 0 | 3 | - | - | 5 |
| *Harderian glands* | (10) | (0) | (0) | (10) | (10) | (0) | (0) | (10) |
| Infiltration, inflammatory cell | 4 | - | - | 1 | 0 | - | - | 1 |
| *Zymbal’s gland* | (0) | (0) | (0) | (0) | (0) | (0) | (0) | (1) |
| Inflammation | - | - | - | - | - | - | - | 1 |
| Adenoma | - | - | - | - | - | - | - | 1 |
| (), no. of animals examined microscopically at this site; -, not applicable; *A. tubingensis*, *Aspergillus tubingensis*; AG, arabinase from non-genetically modified *A. tubingensis* strain GPA41; TOS, total organic solids.  ^†^ Control animals were administered distilled water. | | | | | | | | |
